# Assessing the Efficacy of Pyrolysis–Gas Chromatography–Mass Spectrometry for Nanoplastic and Microplastic Analysis in Human Blood

**DOI:** 10.1021/acs.est.4c12599

**Published:** 2025-01-24

**Authors:** Cassandra Rauert, Nathan Charlton, Angus Bagley, Sarah A. Dunlop, Christos Symeonides, Kevin V. Thomas

**Affiliations:** † Queensland Alliance for Environmental Health Sciences (QAEHS), The University of Queensland, 20 Cornwall Street, Woolloongabba, Queensland 4102, Australia; ‡ Minderoo Centre − Plastics and Human Health, 20 Cornwall Street, Woolloongabba, Queensland 4102, Australia; § Minderoo Foundation, Perth, Western Australia 6009, Australia; ∥ School of Biological Sciences, The University of Western Australia, Perth, Western Australia 6009, Australia; ⊥ Centre for Community Child Health, Royal Children’s Hospital, Parkville, Victoria 3056, Australia

**Keywords:** microplastics, nanoplastics, pyrolysis−gas chromatography−mass spectrometry, matrix interferences, human exposure studies

## Abstract

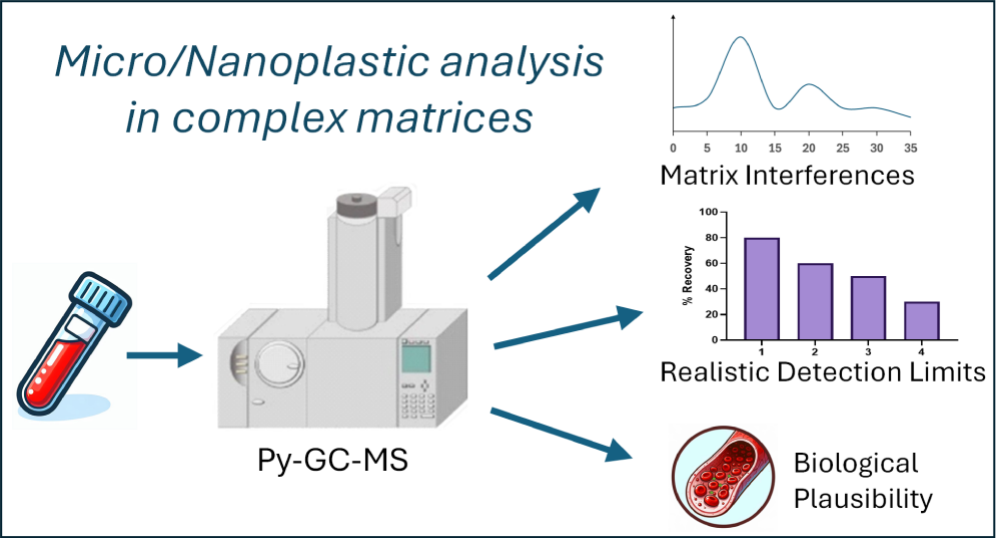

Humans are constantly exposed to micro- and nanosized plastics (MNPs); however, there is still limited understanding of their fate within the body, partially due to limitations with current analytical techniques. The current study assessed the appropriateness of pyrolysis–gas chromatography–mass spectrometry (Py-GC-MS) analysis for the quantification of a range of polymers in human blood. An extraction protocol that reduced matrix interferences (false positives) of polyethylene (PE) and polyvinyl chloride (PVC) was developed and validated. Extraction recoveries ranged 7–109%, although surface-modified polystyrene (carboxylated) increased nanoparticle recoveries from 17 to 52%. Realistic detection limits were calculated for each polymer, accounting for matrix suppression and extraction recovery. These were up to 20 times higher than nominal detection limits calculated with Milli-Q water. Finally, the method was tested with a pilot study of the Australian population. PE interferences were reduced but still present, and no other polymers were above detection limits. It was concluded that Py-GC-MS is currently not a suitable analysis method for PE and PVC in biological matrices due to the presence of interferences and nonspecific pyrolysis products. Furthermore, while it is plausible to detect some polymers in blood, the estimated exposure concentrations needed are approaching the detection limits of the technique.

## Abstract 2

The efficacy of Py-GC-MS analysis of MNPs in biological matrices was assessed, determining that it is not appropriate for certain polymers and may not have the detection limits needed for biologically feasible exposures.

## 1 Introduction

Humans are continuously exposed to micro and nanosized plastics (MNPs) through everyday activities. These exposure pathways include ingestion, inhalation, and potentially dermal exposure for nanosized particles^1,2^ although current understanding on the fate of plastic particles within the body, such as residence time in the circulatory system, uptake in organs, or elimination pathways are still limited. Recently, an increasing number of human biomonitoring studies on MNPs have been reported, aiming to address these knowledge gaps. However, there is a lack of standardization in the field with a range of methods and analytical techniques used. This has resulted in a wide range of reported concentrations and particle sizes which may lack biologically plausibility.

**Figure 1:**
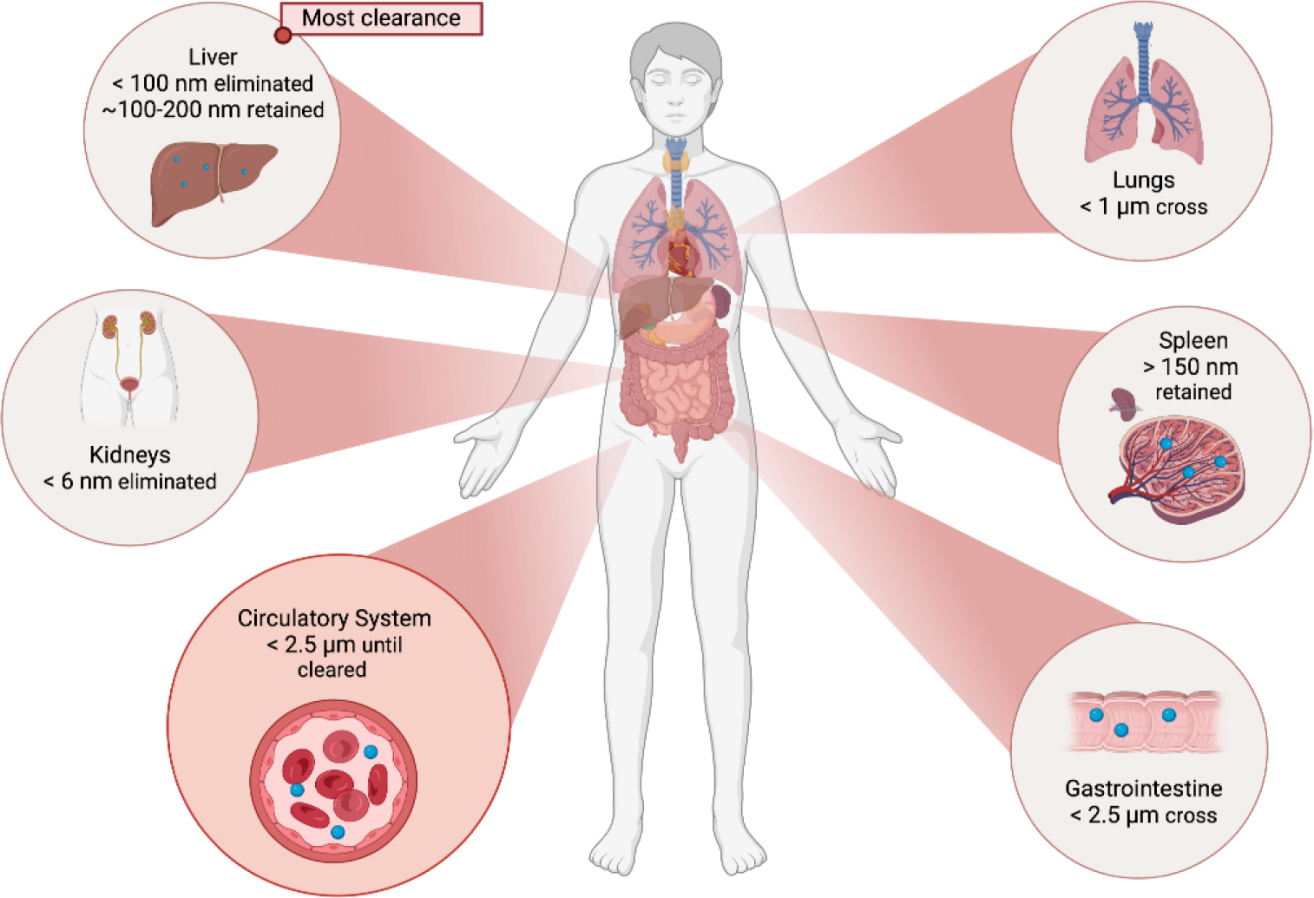
Schematic of biological fate of nanosized and small micron-sized particles. Created in BioRender. [Angus Bagley] (2025) https://BioRender.com/x63r090.

Previous studies on nanosized particles (e.g., particulate matter or particles engineered for pharmaceutical purposes such as drug delivery) can provide some insight on the potential fate of MNPs within the body (Figure 1). Once ingested, particles smaller than 2.5 μm can enter the gastrointestinal tract through endocytosis and enter the circulatory system.^2,3^ Airborne particles smaller than 10 μm can be inhaled through to the terminal branches and alveolar air sacs of human airways^3^ and particles smaller than 1 μm (nanoparticles) have the potential to cross lung tissue barriers and also enter the circulatory system.^2^ As the smallest internal diameter of capillaries are typically ∼7–17 μm,^4^ only very small micron or nanosized particles can be transported through the body. Once in the bloodstream, particles smaller than ∼6 nm are rapidly eliminated through the kidneys via urinary excretion^5,6^ while larger particles can be cleared from circulation by the mononuclear phagocyte system (MPS)^7^ through sequestration in the liver and spleen.^5^ The majority of particle clearance occurs within the liver with an estimated removal of 30–99% of nanoparticles in the bloodstream^5^ although this is dependent on particle properties such as size and charge. Particles that are not taken up by the liver may be cleared by the liver during subsequent passes.^8^ Within the liver, particles >100 nm can be retained long-term within Kupffer cells^6^ whereas smaller particles^5^ can pass into the space of Disse for excretion via the biliary pathway for eventual elimination in the feces.^6^ The spleen is the largest blood filtering organ in the body, eliminating particles >150 nm^9^ with splenic uptake increasing with particle size.^9^ Uptake to spleen macrophages is significantly reduced as compared to Kupffer cells.^8^ Certain medical conditions can increase the presence of barrier cells which work in conjunction with macrophages to increase clearance^9^ or create leaky barriers allowing increased migration.^10^

The uptake and retention of nanoparticles in the body has been shown to be strongly correlated with surface charge, ligand chemistry and size. Nanoparticles used in drug delivery are often coated with polyethylene glycol (PEG) to increase hydrophilicity and neutral surface chemistry, increasing blood circulation half-lives.^7^ Highly cationic/anionic surface charges can absorb proteins to form a protein corona which increases interaction with macrophages,^5,8^ and certain nanoparticles have demonstrated clearance from the bloodstream within a few hours post injection.^7^ Therefore, it is expected that only particles in the small micron or nanosize range will cross biological barriers, and are expected to be cleared from the circulatory system rapidly. These mechanisms will be dependent on health and disease states of the person, and the characteristics of the particle (e.g., size, shape, charge, corona). It is still largely unknown how these factors affect the migration of polymer nanoparticles (nanoplastics) following exposure.

Current human biomonitoring studies that aim to provide information in this area use a range of analytical techniques with the most used being vibrational spectroscopy-based methods. These techniques are marketed to reliably identify particles larger than ∼20 μm in size, or ∼1–5 μm for μ-FTIR and μ-Raman. Therefore, detecting particles that are expected to cross biological barriers is challenging and pushes the detection limits of these instruments. Regardless, recent studies have used these techniques to report MP particles in the human circulatory system ranging in size from 20–184 μm^11^ to 7–3000 μm^12^ and in clearance organs (the liver) from 4–30 μm,^13^ sizes that are often larger than those realistically expected to cross biological barriers into these matrices or even have the ability to circulate through capillary beds. Mass spectrometry-based techniques improve on these size limitations but lose some of the characterization information obtained from spectroscopy-based techniques. Leslie et al.^14^ was the first to use pyrolysis–gas chromatography–mass spectrometry (Py-GC-MS) to report polymers >700 nm in human blood. Since this first study, there has been an exponential increase in the number in human exposure studies on MNPs using thermal decomposition mass spectrometry techniques. Py-GC-MS has been used to report polymers in venous blood, feces, urine, semen, placenta, arterial tissue, arterial plaque, thrombi, gallstone, tumors, bone marrow, testes and vitreous humor (Table S11).^14−29^

However, the use of Py-GC-MS, even as an environmental monitoring tool for MNP pollution, is still in its infancy and these techniques are employed without fully understanding, or assessing, their uncertainties. For example, both spectroscopy and thermal degradation techniques can suffer from interferences within a complex sample,^30−34^ leading to false positives. For Py-GC-MS this uncertainty stems from the method being an indirect analysis technique, as thermal decomposition products (small organic molecules) of a polymer are analyzed, not the polymer itself. This provides the opportunity for endogenous compounds of similar structures to decompose into the same molecules. For example, lipids are a significant matrix interference in the analysis of polyethylene (PE),^31^ thermally decomposing into the same series of alkanes, alkenes and alkadienes as PE, hence will provide a false positive PE detection in samples. These matrix interferences cannot be completely removed during analysis^31^ and need to be assessed and removed during the sample workup stages. Additionally, some polymers such as polyvinyl chloride (PVC) break down into nonspecific molecules, forming a range of common polycyclic aromatic hydrocarbons during pyrolysis. Without a selective pyrolysis product to analyze, the uncertainty around confident identification and quantification increases.

Human exposure studies are the backbone for understanding fate and persistence of target chemicals of concern (including MNPs) within the body. Therefore, it is paramount that robust and reliable biomonitoring data are produced to aid with understanding fate and toxicology and development of well-informed regulations. To this end, the aim of this study was to build on the previous pioneering work in this area and assess the appropriateness of using Py-GC-MS as an analysis technique for identification and quantification of a range of polymers in human blood. Three extraction protocols were assessed for removal of matrix interferences and the final developed method validated against recovery of both nano- and micron-sized polymers, with realistic detection limits determined and the biological plausibility of mass-based concentrations that can be confidently reported with this methodology discussed. Finally, the methodology was tested with a pilot study of blood samples from the Australian population, using the strict QA/QC criteria developed to determine if plastics >300 nm can be identified confidently.

## 2 Materials and Methods

### 2.1 Chemicals

Liquid chromatography grade dichloromethane (DCM, LiChrosolv), gradient grade methanol (LiChrosolv) and Proteinase K (lyophilized, 30 mAnson-U/mg) were purchased from Merck Pty Ltd. (Bayswater, VIC, Australia). Hydrogen peroxide (H_2_O_2_, Emsure, 30%), calcium chloride dihydrate (ReagentPlus grade, ≥99.0%) and tris hydrochloride (reagent grade, ≥99.0%) were purchased from Sigma-Aldrich (Bayswater, VIC). Analytical reagent grade ethanol (100%, undenatured), calcium chloride (fused dihydrate) and sodium carbonate (anhydrous) were purchased through ChemSupply Australia (Gillman, SA). Where possible, reagents were purchased in glass bottles. CREON 10,000 (Abbott Laboratories GmbH, Germany, Mylan), an over the counter pancreatic enzyme replacement medication containing lipase, amylase and protease (10,000, 8000, and 600 Ph Eur units, respectively), was purchased from a local pharmacy in Brisbane, Australia.

Ultrapure water was purified with a Milli-Q system (Millipore, Bedford, USA) and again filtered through a furnaced (500 °C) 0.3 μm glass fiber filter (Advantec, 47 mm GF-75, Labtek, Brendale, QLD) prior to use. Aqueous solutions of reagents were prepared in filtered Milli-Q water as required. Reagents also underwent an additional 0.3 μm filtering step following preparation and before use. Adjustment of all sample and reagent pH was achieved with a saturated sodium carbonate solution and monitored through an OHAUS Starter 300 pH Meter with an attached ST320 pH probe (OHAUS, Melbourne, Victoria).

Nanosphere (NS) solutions of polystyrene (200, 300, 400, 500, 700, 1000 nm) and poly(methyl methacrylate) (400, 740, and 1100 nm) were purchased from Bangs Laboratories, Inc. (Fishers, USA) and nanosphere solutions of carboxylated polystyrene (COOH-PS) were purchased from PolySciences (Taipei, Taiwan). Specific details of the NS solutions are listed in Table S2. Powdered standards of polyethylene terephthalate (PET), polycarbonate (PC), deuterated polystyrene (*d*_5_-PS), 4-fluorinated polystyrene (4-FlPS) and deuterated polyethylene (*d*_4_-PE) were purchased from Polymer Source, Inc. (Dorval, Canada). Polystyrene (PS) and poly(methyl methacrylate) (PMMA) were purchased from Sigma-Aldrich (St. Louis, MO, USA), low-density polyethylene (PE) was purchased from Thermo Fisher Scientific (Scoresby, VIC) and polypropylene (PP) was donated by a plastic manufacturer from Melbourne, Australia (LyondellBasell, VIC).

Glass fiber filters (21 mm, 0.3 μm and 20 mm, 1.0 μm pore size from Advantec (Osaka, Japan) and 47 mm, 0.7 μm pore size from Whatman (Seoul, South Korea)) were furnaced (Nabertherm Muffle Furnace, Model LT 40/11m, Nabertherm GmbH, Lilienthal, Germany) at 500 °C for 8 h prior to use. The 47 mm filters were precut into 21 mm circles with furnaced and DCM rinsed stainless-steel surgical scissors prior to furnacing and use.

### 2.2 Samples

Human ethics for collection of blood for method development and the population pilot study was obtained from the UQ Human Ethics Research Committee (HE001017). Blood used for method development was donated from one participant and this blood was used throughout the method development process for consistency. The pilot study saw collection from 8 participants with samples collected twice: early morning before food intake (fasting) and within an hour after consuming a large meal (nonfasting). One participant donated samples in fasting and nonfasting conditions for 4 days to assess interday variability. Samples were collected in glass 8.5 mL vacutainer tubes containing the anticoagulant acid citrate dextrose, with an eclipse 21G needle (McFarlane Medical and Scientific, Ringwood VIC) creating a direct connection from the draw site to the collection vial, thus avoiding the use of plastic tubing. All samples were stored at −20 °C until analysis. Sampling blanks were prepared by drawing prefiltered Milli-Q water into a glass vacutainer tube (using the 21G needle), storing with the samples, and subsampling for analysis with every batch of blood samples.

### 2.3 Sample Extraction (Final Method)

The final method was chosen after comparison of 3 extraction methods (Section 2.4), with Method 3 chosen due to reduced matrix interferences and improved ease of use (Section 3.1). Frozen blood samples were thawed and mixed via manual agitation of the vacutainer for 30 s. One mL of whole blood was transferred to a 400 mL tall form beaker via a graduated glass pipet. The beaker was immediately capped with a thick aluminum foil lid to minimize potential contamination from atmospheric deposition. Following aliquoting, 10 mL of tris hydrochloride solution (Tris-HCl, 400 mM, 0.3 μm filtered, pH 8) was added, and samples were heated at 60 °C for 1 h to aid denaturing of proteins. After cooling to room temperature, 100 μL of proteinase K solution (1 mg/mL in Milli-Q water, prepared fresh each batch) and 1 mL of calcium chloride solution (5 mM in Milli-Q, filtered at 0.3 μm) was added, and samples were incubated in a Thermoline Orbital Incubator shaker (Thermoline Scientific, Wetherill Park, NSW) at 38 °C for 2 h.

A freshly made 2.5% w/v CREON enzyme (pancrelipase) solution^31^ was prepared with Milli-Q water and pH adjusted to between 8–10. The samples were adjusted to pH 10, 2 mL of the enzyme solution added, and incubated for 3 h at 38 °C. Following incubation, 10 mL of H_2_O_2_ was carefully added in 1 mL aliquots over 8 h (while keeping the pH between 8–10) and the samples allowed to digest at room temperature for a total of 48 h.

Following the digestion, samples were heated to 60 °C on a hot plate and samples were filtered hot using a 13 mm All-Glass Microanalysis Filter Holder with 100 mL reservoir capacity (Micro-Analytix Pty. Ltd., Taren Point, Australia) attached to a 125 mL Vacuum Filtering Side-Arm Flask with threaded side arm (Merck, Bayswater, VIC, Australia). Samples were filtered through a 0.7 μm glass fiber filter, with the beaker rinsed with Milli-Q water and added to the filtering apparatus. The filtrate was then transferred to a fresh, furnaced and solvent rinsed beaker. Ten mL of H_2_O_2_ was added to the reservoir and left in place for 10 min for an additional digestion of the collected particulates on the filter. After H_2_O_2_ removal the filter was then rinsed with 15 mL of water and 15 mL of ethanol with the washes added to the filtrate. The vacuum was left on until the filter was dry and the filter was transferred to an aluminum foil pocket for storage. The filtering process was repeated with the filtrate using a 0.3 μm filter. The filters were inserted to individual 80 μL pyrolysis cups and spiked with 0.1 μg of *d*_5_-PS and 4-FlPS internal standard solutions prior to analysis by Py-GC-MS/MS.

### 2.4 Comparison of Three Extraction Protocols

Initially, three extraction protocols were compared to determine their suitability for ease of use and in removing potential interferences (false positives) from the blood matrix. In Method 1, samples were digested with a mix of CREON enzymes^31^ and H_2_O_2_ before subsequent filtering through 0.7 and 0.3 μm filters. Full method details are described in Text S1. In Method 2, an adaption of the method from Leslie et al.^14^ was trialed which included a proteinase K enzyme digestion before filtration through 0.7 and 0.3 μm filters and a short H_2_O_2_ digestion within the filtering apparatus (Text S2). Thirdly a method that combined the best attributes of the above two methods was developed and is described above (Section 2.3). Unlike Method 2, sodium dodecyl sulfate (SDS) was not added to the Tris-HCl buffer in Method 3 as there was no observed difference in either ease of filtering or level of interferences with or without it. Optimized parameters, such as % ethanol end wash, protein denaturation temperature, and assessment of interferences with and without the CREON enzyme digestion are described in Text S3. To compare the three extraction methods, five 1 mL aliquots of the method development blood and 2 sampling blanks (Milli-Q water) were extracted following each of the three protocols.

### 2.5 Analysis

Identification and quantification of target polymers was performed with a multishot microfurnace pyrolyzer (EGA/PY-3030D) equipped with an autoshot sampler (AS-1020E) (Frontier Lab Ltd., Fukushima, Japan) and coupled to a GC-MS/MS (GC-2030 coupled to a TQ8050 NX MS/MS) (Shimadzu Corporation, Japan), as previously published.^35^ The pyrolyzer unit was operated in double shot mode, utilizing a thermal desorption step where the sample is heated to 300 °C as a first analysis or “shot” to remove the more volatile interferences. The sample was then pyrolyzed at 650 °C to decompose the remaining polymers into their respective pyrolysis products for GC-MS/MS separation and detection. The MS/MS functionality was utilized to reduce background signal and improve detection limits. This was achieved through monitoring the same *m*/*z* ions in each quadrupole with a low energy set for the collision cell (3 eV) to remove background ions (e.g., air and water). The MS/MS was operated in full scan and MRM mode concurrently to retain sample information.^35^ Pyrolysis and GC-MS/MS details are listed in Tables S3 and S4.

Calibration curves were prepared using a low concentration microplastics calibration standard set in CaCO_3_ diluent and containing 12 common polymers (Frontier Laboratories Ltd., Fukushima, Japan). Eight different masses of the calibration powder were weighed into pyrolysis cups, spiked with 0.1 μg of *d*_5_-PS and 4-FlPS internal standards and analyzed. Due to the low concentrations of polystyrene (PS), poly(methyl methacrylate) (PMMA) and polycarbonate (PC) in these calibration standard sets, a second calibration curve was prepared from a 2 mg/mL dissolved solution of these polymers in DCM. Both sets of standards were overlaid to ensure linearity which was deemed acceptable (R^2^ of 0.993–0.999).

### 2.6 Cryomilled Standards

Small particle size polymer standards were prepared from powdered micron-sized standards. Aliquots of each standard were cryomilled (SPEX SamplePrep 6775, Metuchen, NJ) using 4 consecutive runs consisting of 3 cycles of 2 min grinding, 2 min cool time at 10 counts per second. The samples were then sieved through a 25 μm stainless steel mesh using a purpose-built stainless-steel holder with removable mesh disks, and the <25 μm and >25 μm particle size fractions collected. The <25 μm fraction was retained for PS, PMMA, PVC, PC, Nylon-6 and Nylon-6,6. However, PE, *d*_4_-PE, PP and PET could not be cryomilled to particle sizes <25 μm and the >25 μm fraction was retained for these plastics.

### 2.7 QA/QC

All sample preparation was completed within the *Minderoo Plastics and Human Health plastics-minimized laboratory* in a stainless steel biosafety cabinet and stainless steel fume cupboard to minimize background contamination from the laboratory environment.^35^ Cotton lab coats were always worn and the coats were dyed a jade green for visibility of any fibers. Glassware and metalware were subjected to stringent cleaning conditions prior to use. Labware was washed in a laboratory grade Miele Lab Washer PG8583 SS (Thermo Fisher Scientific, Scoresby, VIC) under alkaline conditions at 93 °C. Glassware and glass fiber filters were also furnaced at 500 °C for 5 h in a Nabertherm Muffle Furnace (John Morris Group, Chatswood, NSW). Glassware was wrapped in foil until use where they were rinsed with DCM prior to use. All samples, solvents and other containers were covered with furnaced aluminum foil when not directly used to minimize contamination from atmospheric deposition during sample processing and reagents were prefiltered through a furnaced 0.3 μm filter before use.

Sampling blanks (Milli-Q water subsampled from the glass vacutainer tube) were processed with each batch of samples. Limits of quantification (LOQ) were calculated as the concentration of a peak with a signal:noise ratio of 10:1. Where a polymer analyte was detected in sampling blanks, method detection limits (MDLs) were calculated as the average concentration in the blanks plus three times the standard deviation. If an analyte was not detected in the blanks the LOQ was used in place of the MDL. For comparison, a recovery detection limit (RDL) was also calculated as the LOQ of an extracted blood sample (to include matrix suppression) times the % recovery of that polymer from the recovery tests (Section 3.2) and represents a realistic detection limit of the final method. MDLs, LOQs and RDLs are listed in Table S5. All concentrations in samples were blank corrected (subtraction of mean concentration from the sampling blanks) and all analyte concentrations were internal standard corrected.

## 3 Results and Discussion

### 3.1 Performance Comparison of Three Extraction Protocols

**Figure 2:**
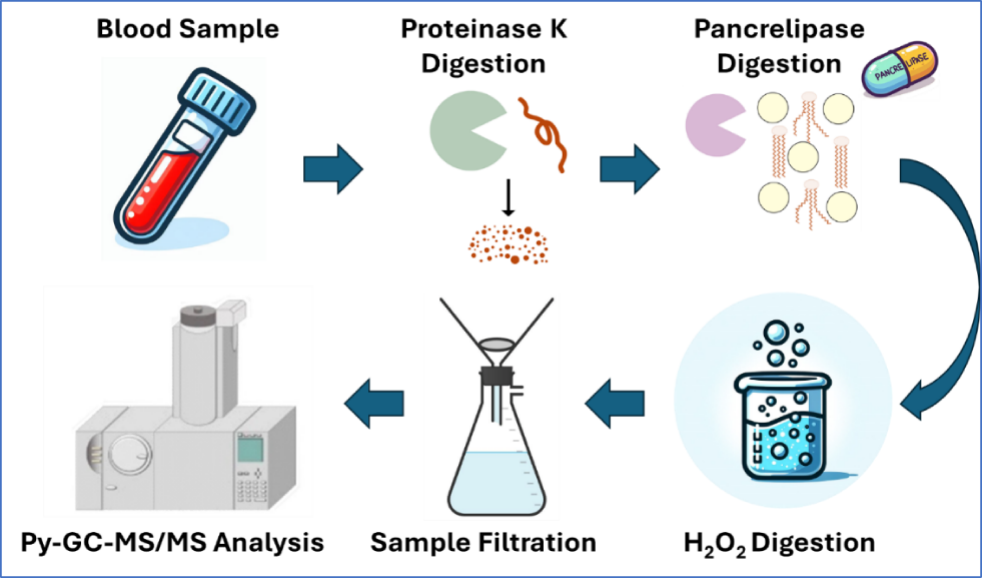
Schematic of final optimized extraction method.

It was observed that Method 1 (CREON only) was difficult to filter, blocking both pore size filters quickly even though the sample was visibly clear following the H_2_O_2_ digestion. Method 2 (proteinase K only) was easier to filter and after the H_2_O_2_ digestion within the filtering apparatus the filter looked visibly clear. Method 3 was the fastest to filter and the addition of the Tris-HCl buffer assisted with controlling pH fluctuations. It was also observed for all three methods, that if the sample was filtered at 1 μm instead of 0.7 μm, then the 0.3 μm filtering step was much slower suggesting significant particulates in the 0.7–1 μm size range. Therefore, the samples were filtered at 0.7 μm for ease of passing the sample through the 0.3 μm filter. A schematic of the final method is shown in Figure 2.

**Figure 3:**
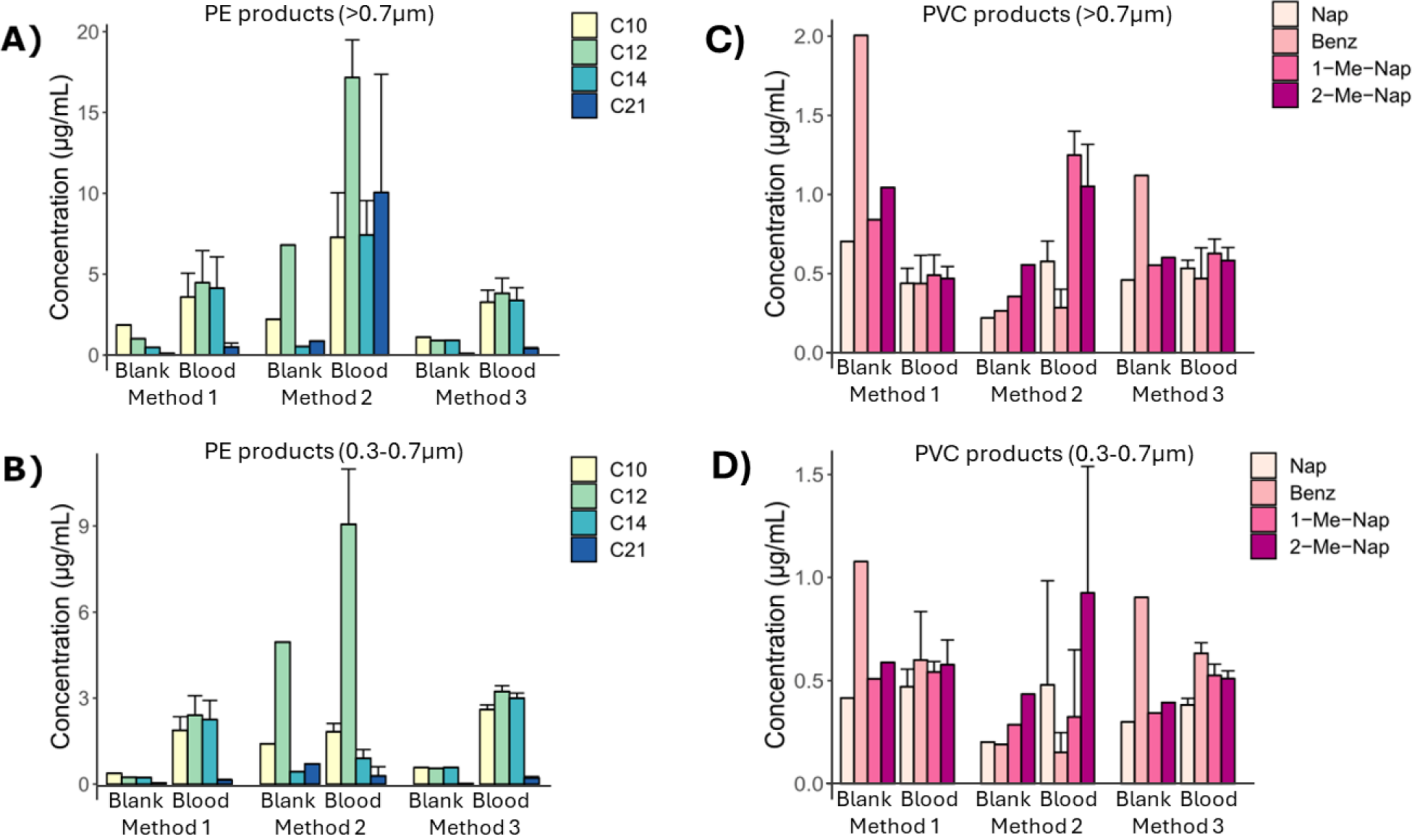
Concentrations calculated using different pyrolysis products of polyethylene (PE) and polyvinyl chloride (PVC), as collected on 0.7 and 0.3 μm glass fiber filters. C10 = C10 alkene, C12 = C12 alkene, C14 = C14 alkene, C21 = C21 alkadiene, Nap = naphthalene, Benz = benzene, 1-Me-Nap = 1-methyl-naphthalene, and 2-Me-Nap = 2-methyl-naphthalene.

Analysis of the filters revealed that PE and PVC pyrolysis products were detected above the MDLs for all three methods (Figure 3, Tables S6 and S7). No other polymers were detected. The ratios of the PE concentration calculated using the 4 monitored PE pyrolysis products were highly variable. This variability suggests that the detected products originated from an interference such as lipids,^31^ rather than from PE itself, as discussed in Section 3.3. Interestingly, the concentrations of the PE pyrolysis products were highest and most variable between replicates using Method 2. This suggests that the CREON enzyme mix, which contains lipase, is necessary to reduce this interference although it does not completely eliminate it.

The PVC pyrolysis products showed greatest variability in the calculated PVC concentrations for Method 2, often showing higher concentrations for either or both of the alkylated naphthalenes. This variability also suggests the presence of a PVC interference and indicates that monitoring the alkylated naphthalenes alone could result in a false positive for PVC in these samples. The calculated concentrations for Methods 1 and 3 were not higher than the blanks, indicating these are not a true PVC result. Previous studies suggest these polycyclic aromatic hydrocarbons can form from pyrolysis of triglycerides and fatty acids.^36^ However, this would need to be investigated with the specific triglycerides present in blood matrices. Due to the ease of filtering and minimization of potential PE and PVC interferences, Method 3 demonstrated the best performance and was used as the final method.

### 3.2 Method Recovery

#### 3.2.1 Nanoplastics

**Figure 4:**
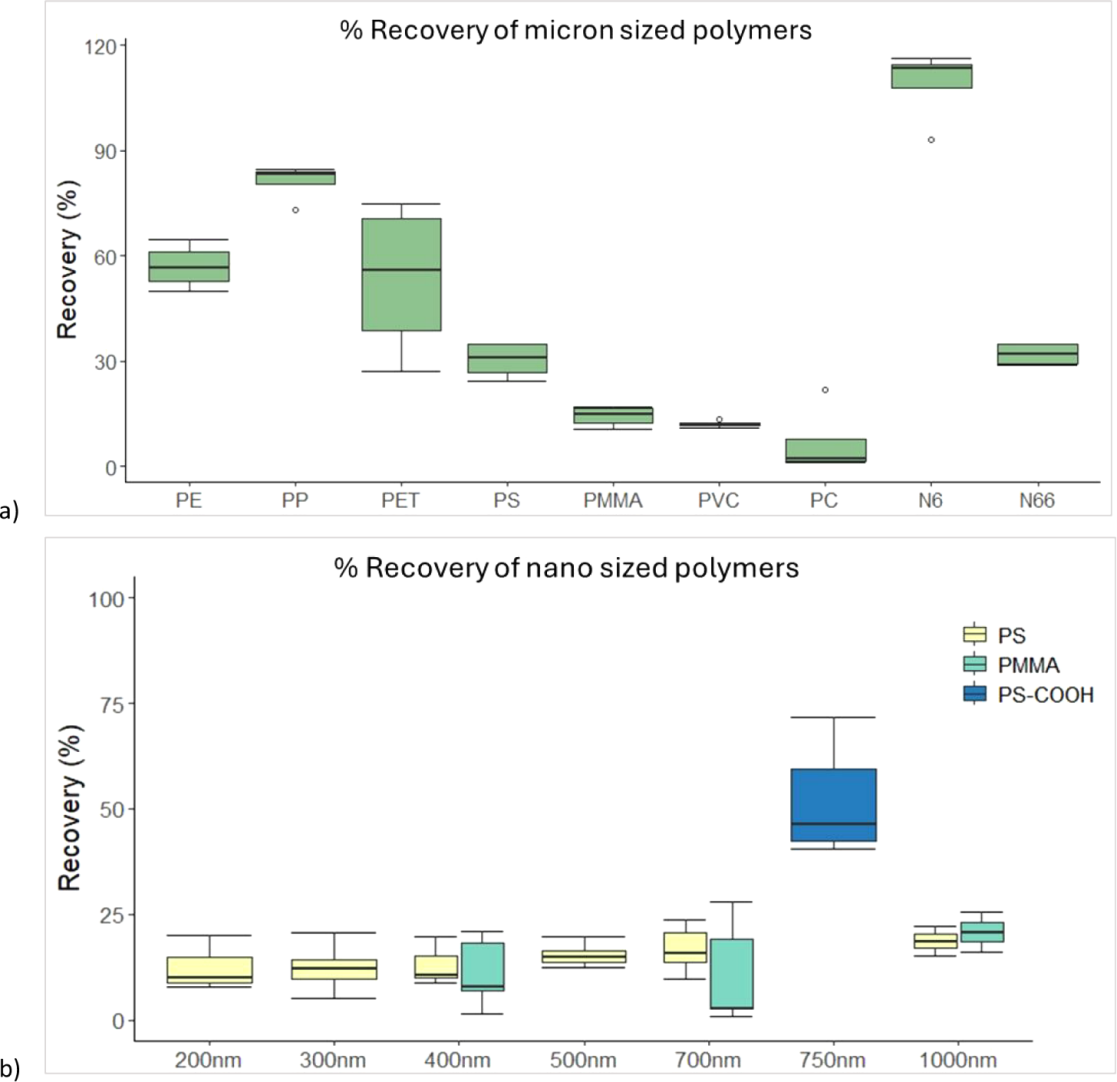
Recovery (%) of (a) micron-sized polymers, (b) nanosized polymers from extracted blood samples. PE = polyethylene, PP = polypropylene, PET = polyethylene terephthalate, PS = polystyrene, PMMA = poly(methyl methacrylate), PVC = polyvinyl chloride, PC = polycarbonate, N6 = Nylon-6, and N66 = Nylon-6,6.

The efficiency of the adopted method for extracting nanosized particles (<1 μm) was evaluated using a range of commercially purchased PS and PMMA nanosphere standards (nanosized standards were not available for other polymers). Stock nanosphere solutions were diluted with filtered Milli-Q to produce a 0.1% dispersion. A 10 μL aliquot, equating to ∼1 μg of polymer and between 1.3 × 10^7^ and 2.5 × 10^9^ particles (Table S2), was spiked into 1 mL of blood with the sample left to equilibrate for 30 min. The blood samples were then extracted using the optimized protocol. The recovery rates (%) for both polymers were low (<20%) but showed a marginal, although not significant (*p* > 0.05), increase with particle size (Figure 4). When recoveries from the filtration step only were tested, the recovery for the 700/740 nm nanospheres was high (81–86%, Table S1) suggesting that losses may be from adsorption to the glassware and transfer. The 200 nm particles were captured on the 0.3 μm filter and particles >400 nm were predominantly captured on the 0.7 μm filter (Table S8). This indicates that aggregation or interaction with matrix material may facilitate the capture of particles on filters with larger pore sizes, demonstrating the potential for this method to capture particles in blood down to 200 nm.

As these nanospheres are virgin plastics and may not represent environmentally relevant nanoplastics, a carboxylated surface modified PS (COOH-PS) was also used to simulate weathered PS particles. A 0.1% dispersion of 750 nm particles was prepared and 50 μL (∼5 μg and 2.7 × 10^7^ particles) spiked into 1 mL of blood (*n* = 7) and extracted. The COOH-PS nanospheres returned a much higher mean recovery of 52% (41–72%, Table S8 and Figure 4), indicating that surface charge or chemistry impacts the behavior of nanoparticles. This suggests that the recoveries of virgin PS and PMMA may not accurately reflect recoveries of nanoplastics that may be present in actual blood samples. This result underscores the importance of evaluating methods with environmentally relevant particles and the need for nanosized standards that model different conditions such as size, shape, charge and (eco)corona.^37^

#### 3.2.2 Microplastics

As nanosized plastics were not available for all the polymers monitored in this study, recovery tests were also conducted using cryomilled micron-sized (>1 μm) powder standards. Each standard (10–30 μg) was weighed and spiked into 1 mL of blood (*n* = 4) and extracted. Recoveries ranged from 7–109% (Figure 4, Tables S9 and S10) with only PE, PP, PET and Nylon-6 showing recoveries >50%. The recoveries of PS and PMMA were higher than those for virgin PS and PMMA nanospheres but remained low (14–30%). PC had the lowest recovery of the study (7%), suggesting it may not be suitable for this method. However, this needs to be assessed further with more environmentally relevant particles. PET recoveries were highly variable between samples, with a relative standard deviation of 42% and recoveries from 11–54% across the three monitored pyrolysis products. Higher average recoveries were observed when using benzoic acid as the quantifying pyrolysis product, and it was therefore chosen as the quantification product in this matrix. Variability in the formation of PET pyrolysis products has been demonstrated previously, in the presence of inorganic matrix components.^38^ The suppressed formation of benzophenone as compared to the benzoic acid may be due to the lack of CaCO_3_ in the samples (as compared to the calibration standards), which facilitates this pyrolytic reaction. However, it is unknown what matrix component is suppressing the formation of the vinyl benzoate, and this variability needs to be further investigated to determine if PET can be confidently reported in blood matrices.

The recovery of PE was also assessed using a deuterated-PE (*d*_4_-PE) micron-sized powder standard, to remove the impact from any potential interferences. Again, 10 μg was spiked into 1 mL of blood (*n* = 4) and extracted. Recoveries were very similar to native PE with a mean of 53%. Brits et al.^29^ also assessed MNP recovery of their blood extraction methodology with recoveries ranging from 73–134% for 6 polymers: PMMA, PP, PS, PE PET, PVC. However, their recovery was assessed using dissolved polymer solutions, and the different interactions of complex media with particles as opposed to dissolved polymers may explain the more variable recoveries in this study.

Two internal standards (IS) were added to all samples (*d*_5_-PS and 4-FlPS), and recoveries were also compared based on the IS used for quantification. Only PS and PMMA had higher recoveries when *d*_5_-PS was used with 30 ± 5 vs 21 ± 3% for PS and 14 ± 3 vs 9 ± 1% for PMMA. Interestingly there was no difference for the PS, COOH-PS or PMMA nanospheres using either *d*_5_-PS or 4-FlPS as the IS. Previous studies have reported matrix induced rearrangement of the *d*_5_-PS monomer^39^ in complex matrices. As this was not observed for theses samples, *d*_5_-PS was chosen as the internal standard for this method.

#### 3.2.3 Recovery Detection Limits (RDL)

To determine a realistic detection limit, or a concentration that could be confidently reported in matrix, an RDL was calculated. To include the influence of signal suppression from matrix, this consisted of the concentration of a peak with a signal:noise ratio of 1:10 in an extracted blood sample. This concentration was then corrected by the method recovery determined for each polymer from the MP recovery tests. This was assumed to provide a more realistic blood concentration that could be confidently reported using the current methodology, assuming absence of background contamination or interferences. This calculation is described in eq 1.
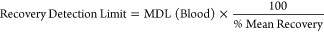
1

A comparison of the calculated RDL and the LOQs calculated from the sampling blanks is shown in Table S5. The pyrolysis products for PP, Nylon-6 and the PS (specifically PS trimer) were the only products not affected by the presence of matrix. For the other polymers, the RDL increased over the LOQ from 3 (PE) up to 65 times (PET). This resulted in a required PET concentration of 2.4 μg/mL in blood to allow the detection of benzoic acid and 12 μg/mL to detect the vinyl benzoate product of PET. It is noted that these samples were analyzed collecting MRM and Scan data concurrently, and running the samples in just MRM or SIM mode may improve sensitivity, however the ability to retrospectively assess samples using a range of pyrolysis products would be lost, and thus assessment of potential interferences would also be lost.

The calculated RDLs were assessed for the biological plausibility of these concentrations occurring in an average human’s circulatory system. Considering the human body has a volume of blood of ∼5 L, a PET concentration of 12 μg/mL would equate to 60 mg of plastic within the circulatory system. There is limited data on absorption efficiency of nanoplastics through the gastrointestinal tract. Previous studies have estimated an absorption efficiency of up to 1.7% for 50 nm PS nanoparticles.^40^ Additionally, there is currently no information on circulation half-lives of environmentally relevant nanoplastics, but previous studies have shown 200 nm spherical poly(ε-caprolactone) (PLA) particles have a half-life of 12 h.^41^ Assuming this efficiency and circulation half-life is maintained for the larger 300–700 nm particles captured by this protocol (which is likely an overestimate), the participant would have to be exposed (ingested/inhaled) to at least 7 g of PET, within this size-range, every day to maintain a steady state concentration. This is almost two credit cards worth of plastic and is highly unrealistic. For the polymers with lowest RDLs (PP, PMMA, Nylon-6, Nylon-6,6: 0.01–0.02 μg/mL), an ingested/inhaled plastic mass of at least 6 mg per day is needed, which may be feasible. Of course, there are many assumptions with this calculation due to unknowns with biological transport, efficiency and bioavailability which can be impacted by individual biology, physiology of the GI tract, age, and disease state.^42^

### 3.3 Polymer Concentrations in Human Blood

A pilot study was conducted as the final validation step. All collected samples were analyzed in triplicate (1 mL aliquots). The pyrolysis products of PMMA, PET, PVC, PC, Nylon-6 and Nylon-66 were below MDLs in every sample, Supporting Information file 2. Three samples had PP concentrations above the MDLs (3.1 to 4.9 μg/mL). However, these concentrations are exceedingly high, and in each case only detected in one of the triplicates analyzed, suggesting sporadic background contamination. Based on the above exposure estimations, a PP concentration of 5 μg/mL would equate to 25 mg of plastic within the circulatory system and the participant would have to be exposed to 3 g of PP every day. This quantity of PP exposure is biologically implausible for the average human, and combined with detection not being replicated across triplicates, indicates contamination as a more likely source.

The PS dimer and trimer were detected together in 2 samples, but only in 1 of the triplicates in all cases, indicating potential contamination due to lack of reproducibility. Blood for participant #5 had PS pyrolysis products in the 0.3–0.7 μm size range in all three triplicates of the fasting sample. The dimer was above MDLs (0.05–0.22 μg/mL), while the trimer was below the MDL (0.019–0.52 μg/mL) making it difficult to confirm the presence of PS by both pyrolysis products. A PS concentration of 0.15 μg/mL (average of the triplicates) would equate to ∼750 μg within the participants circulatory system or exposure to 88 mg per day. Assuming this estimated PS exposure is feasible, the current evidence suggests only an indicative detection of PS in blood. More replicates of this sample need to be analyzed to eliminate the possibility of contamination. Additionally, confirmation with another, noncorrelated technique is needed e.g., imaging with spectroscopy-based identification. Although it is noted that analytical options are limited for small particle sizes (300–700 nm).

#### 3.3.1 PE and PVC Interferences

The PVC pyrolysis products were detected in all Milli-Q blanks, and concentrations in all samples were below MDLs (0.9–4.2 μg/mL, Supporting Information file 2). In contrast, the PE pyrolysis products were detected in every sample, with at least one product above the MDL. Our previous research established a quality control framework to improve confidence in reporting PE concentrations and avoid reporting false positives due to interferences.^31^ This framework includes comparing the PE concentration calculated from the 4 monitored pyrolysis products. If the ratio of the concentration calculated with the C10 alkene to the concentration calculated with the C12 alkene, C14 alkene or C21 alkadiene differs by more than a factor of 2, then an interference is likely present. In these samples (not blank corrected) the ratio ranged from 0.02–4.1. To further investigate, we assessed 21 different PE pyrolysis products, including alkanes, alkenes and alkadienes of chain length C10, C12, C14, C18, C19, C20 and C21. Figure S2 shows the ratio of the PE concentration calculated from each pyrolysis product to the concentration calculated from the C10 alkene, highlighting the high variability in these ratios.

**Figure 5:**
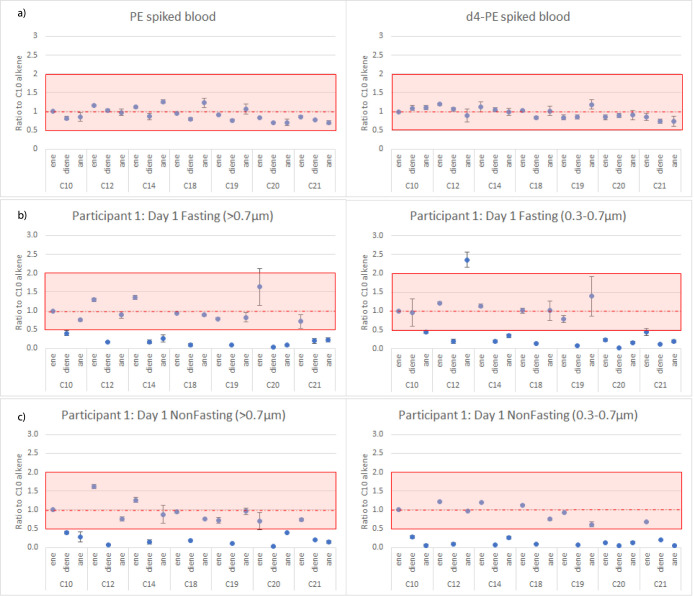
Ratios of PE concentrations calculated using different pyrolysis products of PE to the calculated concentration using the C10 alkene. Positive controls (blood spiked with either *d*_4_-PE or PE) are shown in (a), a fasting sample from participant #1 in (b) and a nonfast sample from the same participant in (c). All graphs from all samples are in Figure S2. Shaded boxes indicate the “acceptable” ratio range for PE identification.

To also compare with a positive control, the same pyrolysis products were extracted from blood samples spiked with PE and *d*_4_-PE in the method recovery validation experiments, Figures 5 and S2. These spiked samples showed a consistent ratio with the C10 alkene, ranging from 0.7 to 1.2. This further confirms that the high variability in calculated PE concentrations of the pyrolysis products in the pilot study samples indicates significant interferences. In these samples similar calculated PE concentrations were detected between size ranges (>0.7 μm or 0.3–0.7 μm) and there was also no trend in pattern between the fasting and nonfasting samples across participants, despite triplicates being very consistent. Participant #2 had higher concentrations in the nonfasting sample, participants #4 and #7 had higher concentrations in the fasting samples, with no difference for the other participants.

The significant interference in the PE analysis and the inability to successfully remove it highlights the need for caution in human exposure studies to avoid misreporting PE concentrations. Previous studies using Py-GC-MS as an analysis tool for human biomonitoring have primarily reported elevated concentrations of PE and PVC, (0.7–7.1 μg/mL and 0.09–22,000 μg/g for PE; 0.2–25 μg/mL and 0.05–5,000 μg/g for PVC, Table S11) in internal matrices. However, it is unclear if these studies have considered the possibility of matrix interferences, which should be a primary consideration in future studies.

### 3.4 Considerations for Future Studies

#### 3.4.1 Detection Limits and Biological Plausibility

For the analysis of complex matrices, such as in human biomonitoring, it is essential to assess realistic detection limits and determine if these concentrations are biologically feasible within the investigated matrix. Recovery validation experiments are crucial to assess the robustness of current methods and provide greater confidence in the quality of the reported data. As demonstrated in the current study, certain pyrolysis products can be influenced by matrix suppression and lower recoveries, increasing realistic detection limits by 20 times over MDLs calculated from blanks. To address this, more reference standards of environmentally relevant nanosized plastics are needed.

#### 3.4.2 Interferences

Validating methods must include an investigation of potential matrix interferences, regardless of the analysis technique used. In the current study advanced measures were implemented to remove these interferences including extensive digestion protocols and Py-GC-MS analysis in double shot mode. Despite these efforts trace levels of interferences persist. Therefore, the authors suggest that Py-GC-MS is not an appropriate technique for reporting PE and PVC in biological matrices using current methodologies.

#### 3.4.3 Background Controls

The importance of appropriate quality control measures to reduce, eliminate and monitor background contamination in MNP studies cannot be overstated. As observed in the current study, even with the extreme protocols employed (e.g., extraction within the *Minderoo Plastics and Human Health plastics-minimized laboratory*,^35^ dedicated washing/furnacing procedures for all equipment), sporadic contamination can still occur. It is crucial to address this in all studies.

The current study thoroughly assessed Py-GC-MS as an analytical technique for confidently reporting MNPs in human matrices, specifically blood. Several limitations were identified, leading to the conclusion that Py-GC-MS is not currently a suitable technique for identifying PE or PVC due to persistent interferences that are reduced by enzymatic digestion but not fully removed. The method is also not suitable for PET due to matrix suppression and high detection limits. Py-GC-MS may currently be suitable to detect PP, PMMA, PS, Nylon-6 or Nylon-66 in blood matrices, but only at the upper end of concentrations that might be biologically feasible if interferences and background contamination are reduced/removed. It is noted that certain medical conditions such as implantation of polymer materials,^43^ or the use of intravenous lines^44^ may be a more feasible pathway for NPs to enter the bloodstream in detectable concentrations. This should be a priority area for future research.

## Supporting Information Available

Descriptions of three tested extraction methodologies, details of nanoparticle standards and Py-GC-MS conditions, details on blanks and calculated detection methods, tables of calculated PE and PVC interferences, method recoveries from micro- and nanosized standards, table of MNP concentrations previously reported using Py-GC-MS, and poymer concentrations in blood samples and calculated PE interferences (PDF)
Plastic concentrations and PE interfaces (XLSX)
