# Supplementary material for: Assessing the Efficacy of Pyrolysis–Gas Chromatography–Mass Spectrometry for Nanoplastic and Microplastic Analysis in Human Blood
Source: Environ Sci Technol. 2025 Jan 24;59(4):1984–94. doi: 10.1021/acs.est.4c12599 (PMC11800385; doi:10.1021/acs.est.4c12599)
Supplement: Supplementary file 1 — es4c12599_si_001.pdf [file es4c12599_si_001.pdf]

Supplementary Information

Assessing the efficacy of pyrolysis gas chromatography mass spectrometry for Nanoplastic and Microplastic analysis in human blood.

Cassandra Rauert[1,2*], Nathan Charlton[1,2], Angus Bagley[1,2], Sarah A. Dunlop[3,4], Christos Symeonides[3,5], Kevin V. Thomas[1,2]

[1]Queensland Alliance for Environmental Health Sciences (QAEHS), The University of Queensland, 20 Cornwall Street, Woolloongabba, 4102, QLD, Australia

[2]Minderoo Centre – Plastics and Human Health, 20 Cornwall Street, Woolloongabba, 4102, QLD, Australia

[3]Minderoo Foundation, Perth, 6009, WA, Australia

[4]School of Biological Sciences, The University of Western Australia, Perth, 6009, WA, Australia

[5]Centre for Community Child Health, Royal Children's Hospital, Parkville, 3056, VIC, Australia

*corresponding author: c.rauert@uq.edu.au

Summary: 18 pages containing 3 text, 11 tables and 2 figures as outlined in the Table of Contents

Table of Contents:

Text S1: Extraction Method 1 details (Initial test method)

Text S2: Extraction Method 2 details (Adapted from Leslie et al, 2022 [1])

Text S3: Details of parameter optimisation of Method 3

Table S1: Ethanol recovery test results – concentrations collected on 0.7µm filter

Table S2: Details of nanoparticle solutions.

Table S3: Pyrolysis gas chromatography mass spectrometry analysis conditions

Table S4: Pyrolysis products monitored and MS conditions

Table S5: Concentrations in process blanks, Method Detection Limits (MDLs), Limits of Quantification (LOQs) and Recovery Detection Limit (RDL)

Table S6: Three method comparison of interferences: >0.7 µm particle sizes

Table S7: Three method comparison of interferences: 0.3 - 0.7 µm particle sizes

Table S8: PS and PMMA and COOH-PS nanosphere recoveries from spiked blood samples

Table S9: Microplastic recoveries from spiked blood samples

Table S10: Recoveries of PE and d_4-PE as calculated using 21 different pyrolysis products

Table S11: Concentrations of MNPs reported in previous human exposure studies

Figure S1: Concentrations of PE and PVC interference, with or without CREON® enzyme digestion

Figure S2: Ratios of calculated concentrations of 21 different pyrolysis products to the C10 alkene

Text S1: *Details of extraction Method 1*

Our previously reported extraction method for mid-high lipid food samples [2] was modified. In short, 1 mL of 5% bile salts solution (Signa Aldrich, Bayswater, AUS) prepared in MilliQ water was added to 1 mL of blood. The sample was vortexed for 30 seconds then 2 mL of freshly prepared 2.5% CREON® 10,000 enzyme solution (Abbott Laboratories GmbH, Germany, Mylan) prepared in MilliQ water was added. The sample was again vortexed to mix, the pH adjusted to between 8-10 with saturated sodium carbonate solution, and samples incubated at 38 °C for 2 hours to facilitate the enzyme digestion. Following incubation, an excess of hydrogen peroxide was slowly added (~10 mL) over 8 hours, keeping the solution between pH 8 – 10 with saturated sodium carbonate. The sample was then sequentially filtered through a 21 mm diameter 0.7 µm and 0.3 µm glass fibre filter. The filters were dried, placed in pyrolysis cups, spiked with internal standards and analysed with Py-GC-MS/MS.

Text S2: *Details of extraction Method 2 (Adapted from Leslie et al, 2022 [1])*

1 mL of blood was subsampled and 15 mL of 400 mM Tris-HCl buffer (with 0.5% SDS) was added, samples were heated in an incubator at 60 °C for 1 hour to denature proteins, then 100 µL of 1 mg/mL Proteinase K solution and 1 mL of 5 mM calcium chloride solution added. The sample was incubated at 38 °C for 2 hours. The samples were then heated at 60 °C for 20 mins and filtered through 0.7 µm and 0.3 µm filters. Whist still within the filtering apparatus, the filters were covered with ~10 mL of hydrogen peroxide which was allowed to sit for ~15 min. The hydrogen peroxide was removed from the sample by vacuum filtration then 15 mL of MilliQ water was filtered through the filter to remove any traces of hydrogen peroxide. The filters were dried, placed in pyrolysis cups, spiked with internal standards and analysed with Py-GC-MS/MS.

Text S3: *Parameter optimisation for Method 3*

Ethanol wash

A 0.3 µm filter was placed inside the filtering apparatus, spiked with 10 µL of a 1:1000 dispersion of 700 nm PS and 740 nm PMMA in water (equating to a mass of ~1 µg/injection) and the sample washed with either 100% MilliQ, 20% ethanol in MilliQ, 50% ethanol in MilliQ or 100% ethanol. 5 replicates were tested for each wash solution. The recoveries increased with the percentage of ethanol with mean recoveries of PS and PMMA ranging 39 – 81% and 57 – 86% respectively. Therefore a 100% ethanol wash was adopted as the last step for all filtrations in the final method.

Table S1: % Recovery of 700/740 nm PS/PMMA nanospheres collected on 0.7 µm filter, using various %ethanol wash solvents. Recoveries are calculated with both the d_5-PS and 4-FlPS internal standards for comparison.

		d_5-PS		4-FlPS	
		PS 700nm	PMMA 740nm	PS 700nm	PMMA 740nm
0% Ethanol (100% water)	#1	52	71	43	59
	#2	38	61	33	54
	#3	30	62	29	59
	#4	45	45	36	35
	#5	29	47	24	39
	**AVG**	**39**	**57**	**33**	**49**
20% Ethanol	#1	46	68	41	61
	#2	56	79	47	67
	#3	37	49	31	41
	#4	42	70	32	54
	#5	36	44	32	39
	**AVG**	**43**	**62**	**37**	**52**
50% Ethanol	#1	51	86	41	69
	#2	53	73	46	64
	#3	47	66	41	59
	#4	54	58	41	44
	#5	83	90	62	67
	**AVG**	**57**	**75**	**46**	**61**
100% Ethanol	#1	76	95	75	95
	#2	69	78	58	66
	#3	67	67	59	60
	#4	103	101	76	74
	#5	89	88	75	75
	**AVG**	**81**	**86**	**69**	**74**

Protein denaturation

Protein denaturation prior to digestion with Proteinase K was tested at different temperatures [3]. Triplicate 1 mL blood samples were extracted following the final method protocol with heating for 1 hour at either 60, 80 or 100 °C. There was no observable difference between the 60 and 80°C samples, however the 100 °C samples turned a darker colour and were difficult to filter. The background in the Py-GC-MS/MS analysis was higher for the 100 °C, and the 0.7 µm filter had a higher background of PE pyrolysis products (i.e. the interference was increased at this temperature). As there was no difference between 60 and 80°C, 60°C was used as the denaturation temperature in the final experiments.

CREON digestion

The final method was tested with and without the addition of the CREON® enzyme digestion step with 1 mL blood samples extracted in triplicate. The PE interferences on the 0.3 µm filter was reduced with the additional enzyme digestion, although only the change in the C12 alkene was significant (students t-test, $p = 0.01$). Additionally, the PVC markers were also significantly reduced on both the 0.7 and

0.3 µm filters (p = 0.004 to 0.03) with the additional enzyme digestion. Benzene was not significantly different between the samples, but elevated concentrations were detected in the blank and samples which likely masked any differences in interferences. It was also noticed that the background noise level of the pyrogram was reduced for both the 0.7 and 0.3 µm filters when CREON® was employed. Therefore, the additional enzyme digestion step was determined to be necessary.

Figure S1: Calculated concentrations of PE and PVC using different pyrolysis products in blanks (water) and blood samples extracted with Method 3, either with or without CREON® enzyme mix. Due to the variability in the ratios, these concentrations were classed as an interference, not a true result of PE or PVC.

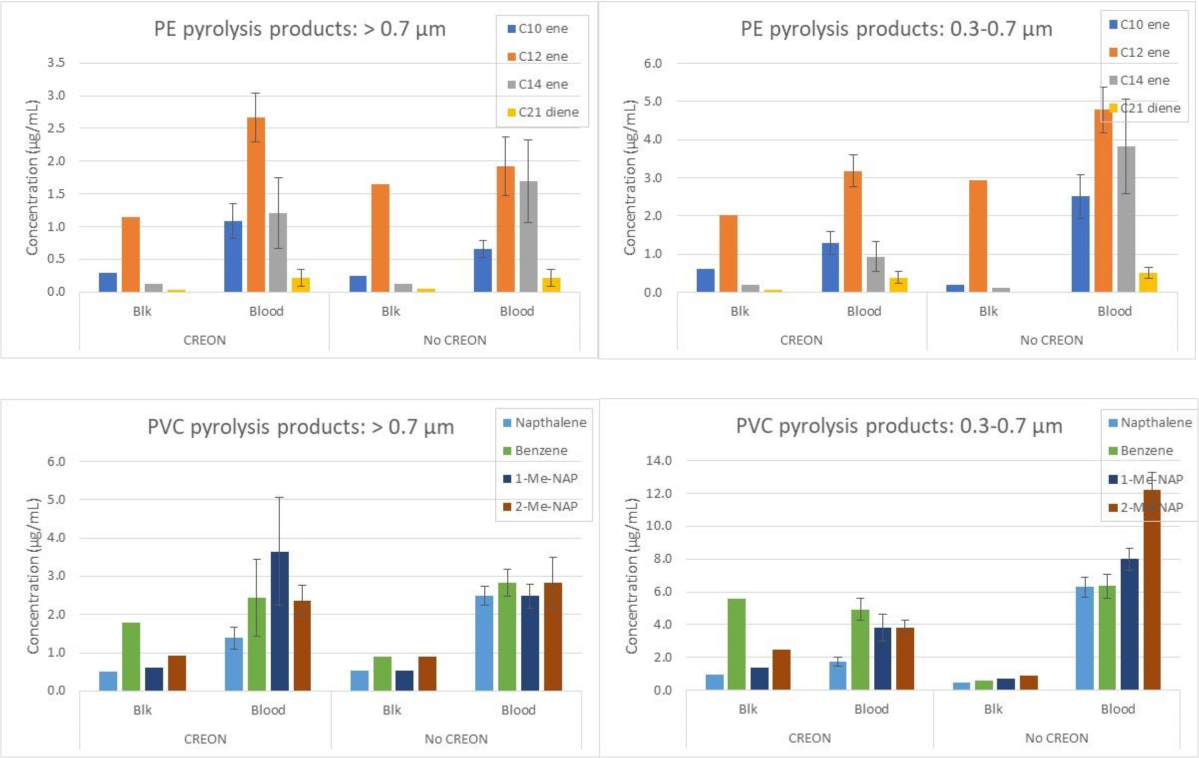

Table S2: Characteristics of nanoparticle solutions including size and number of particles per mL and the calculated number of particles added to blood samples in the method recovery tests.

Polymer	Name	Size (nm)	Particles/mL in Stock	Particles added in recovery tests
PMMA	400	400	2.63×10^{12}	2.63×10^{8}
	740	737	4.20×10^{11}	4.20×10^{7}
	1100	1100	1.26×10^{11}	1.26×10^{7}
PS	200	194	2.52×10^{13}	2.52×10^{9}
	300	308	6.23×10^{12}	6.23×10^{8}
	400	397	2.83×10^{12}	2.83×10^{8}
	500	503	1.46×10^{12}	1.46×10^{8}
	700	710	5.20×10^{11}	5.20×10^{7}
	1000	1040	1.63×10^{11}	1.63×10^{7}
COOH-PS	750	750	1.08×10^{11}	2.70×10^{7}

Table S3: Pyrolysis gas chromatography mass spectrometry analysis conditions

Equipment	Parameters	Settings
Micro-furnace Pyrolyzer (EGA/PY-3030D)	First-shot furnace temperature (thermal desorption)	Ramped; 100 °C → 20 °C /min → 300 °C (1 min)
	Second-shot furnace temperature (pyrolysis)	650 °C
	Interface temperature	320 °C
	Pyrolysis time	12 s (0.20 min)
GC conditions	Model	QP2010 Plus GC-MS (Shimadzu Corporation, Japan)
	Column	Ultra-Alloy® 5 capillary column (30 m, 0.25 mm I.D., 0.25 µm film thickness) (Frontier Lab)
	Injector port temperature	300 °C
	Column oven temperature program	40 °C (2 min) → (20 °C /min) → 320 °C (14 min)
	Injector mode	Split 5:1 or 200:1
	Carrier gas	Helium, 1.0 mL/min, constant linear velocity
MS conditions	Ion source temperature	250 °C
	Ionization energy	Electron ionization (EI); 70 eV
	Scan range	40 to 600 *m/z*
	CID energy<br>Q1 Resolution<br>Q3 Resolution	3 eV<br>Unit<br>High

Table S4: Details on pyrolysis products monitored and MS conditions

Polymer	Abbr.	Pyrolysis Product/s	MRM transitions	Retention Time (min)
Polyethylene	PE	C10-alkene<br>C12-alkene<br>C14-alkene<br>C21-alkadiene<br><br>C10-alkadiene<br>C10-alkane<br>C12-alkadiene<br>C12-alkane<br>C14-alkadiene<br>C14-alkane<br>C18-alkadiene<br>C18-alkene<br>C18-alkane<br>C19-alkadiene<br>C19-alkene<br>C19-alkane<br>C20-alkadiene<br>C20-alkene<br>C20-alkane<br>C21-alkene<br>C21-alkane	**83>83**, 111>111<br>**83>83**, 111>111<br>**83>83**, 111>111<br>**82>82**, 69>69, 96>96<br><br>**81>81**, 67>67, 110>110<br>**85>85**, 98>98, 113>113<br>**81>81**, 67>67, 110>110<br>**85>85**, 98>98, 113>113<br>**81>81**, 67>67, 110>110<br>**85>85**, 98>98, 113>113<br>**81>81**, 67>67, 110>110<br>**83>83**, 111>111<br>**85>85**, 98>98, 113>113<br>**81>81**, 67>67, 110>110<br>**83>83**, 111>111<br>**85>85**, 98>98, 113>113<br>**81>81**, 67>67, 110>110<br>**83>83**, 111>111<br>**85>85**, 98>98, 113>113<br>**83>83**, 111>111<br>**85>85**, 98>98, 113>113	6.54<br>8.27<br>9.75<br>13.74<br><br>6.46<br>6.58<br>8.21<br>8.35<br>9.70<br>9.80<br>12.18<br>12.21<br>12.25<br>12.72<br>12.75<br>12.78<br>13.24<br>13.27<br>13.29<br>13.78<br>13.81
Polypropylene	PP	2,4-dimethyl-1-heptene	70>70, **126>126**	4.92
Polystyrene	PS	3-butene-1,3-diyldibenzene (Dimer)<br>5-hexene-1,3,5-triyltribenzene (Trimer)	91>91, **130>130**, 208>208<br>**91>91**, 117>117, 207>207	11.95<br>15.53
Poly methyl methacrylate	PMMA	Methyl methacrylate	69>69, **100>100**	3.20
Polyvinyl chloride	PVC	Naphthalene<br>Benzene<br>1-Methyl-Naphthalene<br>2-Methyl-Naphthalene	102>102, **128>128**<br>52>52, **78>78**<br>115>115, 141>141, **142>142**<br>115>115, 141>141, **142>142**	8.33<br>2.81<br>9.33<br>9.20
Polyethylene terephthalate	PET	Vinyl benzoate<br>Benzoic acid<br>Benzophenone	51>51, 77>77, **105>105**<br>77>77, **122>122**<br>77>77, 105>105, **182>182**	8.01<br>8.05<br>11.4
Polycarbonate	PC	Bisphenol A	119>119, **213>213**	14.29
Nylon-6	Nylon-6	e-caprolactam	**113>113**, 55>55, 85>85	8.78
Nylon-6,6	Nylon-6,6	Cyclopentanone	**84>84**, 55>55, 41>41	4.21
d_5-Polystyrene	d_5-PS	d_5-styrene monomer	82>82, 108>108, **109>109**	5.47
4-Fluoro-Polystyrene	4-FlPS	4-Fl-styrene monomer	75>75, 96>96, **122>122**	5.55

Table S5: Concentrations in process blanks analysed with population study blood samples. Calculated Method Detection Limits (MDLs) are the average concentration in blanks + 3 times standard deviation. Calculated Limits of Quantification (LOQs) are the concentration of a peak with a S:N ratio of 10:1 in blanks. Where an analyte was not detected in the blank, ½ LOQ was inserted to calculate average concentrations and standard deviations. Recovery Detection Limit (RDL) is the lowest concentration that can be quantified in a blood sample taking into consideration suppression from matrix (blood) background and recoveries of individual polymers (and assuming no interference/background contamination is present).

	PP	PS		PMMA	PET			PC	Nylon-6	Nylon-6,6	PE				PVC			
	DiMe-Hep	Dimer	Trimer	MMA	Benzoic Acid	Vinyl Benzoate	Benzo-phenone	BPA	e-Cap	Cyclo-pentanone	C10 ene	C12 ene	C14 ene	C21 diene	Nap	Benz	1-Me-Nap	2-Me-Nap
LOQ	*0.027*	*0.013*	*0.003*	*0.003*	*0.13*	*0.24*	*0.027*	*0.003*	*0.007*	*0.003*	0.057	0.053	0.050	0.047	0.003	0.003	0.003	0.003
Batch 1 – 0.7 µm	1.72	<0.013	<0.003	<0.003	<0.13	<0.24	<0.027	1.11	0.02	0.07	0.63	0.65	0.63	<0.047	0.24	0.88	0.11	0.41
Batch 1 – 0.3 µm	0.05	<0.013	<0.003	<0.003	<0.13	<0.24	<0.027	3.72	<0.007	0.05	1.00	1.42	1.23	<0.047	0.68	0.18	0.30	0.59
Batch 2 – 0.7 µm	2.08	<0.013	<0.003	<0.003	<0.13	<0.24	<0.027	<0.003	<0.007	0.07	0.69	1.04	0.18	<0.047	0.83	0.81	0.28	0.97
Batch 2 – 0.3 µm	0.05	<0.013	<0.003	<0.003	<0.13	<0.24	<0.027	<0.003	0.03	0.06	0.43	0.46	0.38	<0.047	0.54	0.80	0.24	0.53
Batch 3 – 0.7 µm	0.57	<0.013	<0.003	<0.003	<0.13	<0.24	<0.027	<0.003	<0.007	0.11	0.79	1.00	0.84	0.09	0.42	1.15	0.15	0.64
Batch 3 – 0.3 µm	0.05	<0.013	<0.003	<0.003	<0.13	<0.24	<0.027	<0.003	<0.007	0.13	0.38	0.51	0.51	<0.047	0.35	1.46	0.21	0.75
Batch 4 – 0.7 µm	0.31	<0.013	0.004	<0.003	<0.13	<0.24	<0.027	2.43	0.03	<0.003	0.70	<0.053	0.89	0.06	0.29	0.56	<0.003	<0.003
Batch 4 – 0.3 µm	0.07	<0.013	0.004	<0.003	<0.13	<0.24	<0.027	0.73	0.03	<0.003	0.45	0.54	0.48	0.03	0.12	0.24	<0.003	<0.003
Batch 5 – 0.7 µm	0.28	<0.013	0.045	0.23	<0.13	<0.24	<0.027	0.31	0.03	0.11	0.47	0.70	0.79	<0.047	0.89	3.58	0.68	1.39
Batch 5 – 0.3 µm	0.04	<0.013	0.003	<0.003	<0.13	<0.24	<0.027	0.02	0.02	0.11	0.49	0.82	0.59	<0.047	0.70	2.14	0.55	1.19
Average	*0.52*	*n.a.*	*0.007*	*0.024*	*n.a*	*n.a*	*n.a*	*0.83*	*0.019*	*0.069*	*0.60*	*0.72*	*0.65*	*0.035*	*0.51*	*1.18*	*0.25*	*0.65*
Standard Deviation	*0.75*	*n.a.*	*0.014*	*0.072*	*n.a*	*n.a*	*n.a*	*1.28*	*0.014*	*0.044*	*0.20*	*0.38*	*0.30*	*0.023*	*0.26*	*1.02*	*0.22*	*0.45*
**MDL**	***2.78***	***0.013***	***0.047***	***0.239***	***0.13***	***0.24***	***0.027***	***4.66***	***0.060***	***0.20***	***1.19***	***1.86***	***1.54***	***0.10***	***1.29***	***4.25***	***0.91***	***2.01***
**RDL**	***0.02***	***0.11***	***0.003***	***0.02***	***2.37***	***12.1***	***1.76***	***0.04***	***0.007***	***0.013***	***0.15***	***0.14***	***0.18***	***0.21***	***0.05***	***0.04***	***0.12***	***0.10***

Abbreviations: DiMe-Hep = 2,4-dimethyl-1-heptene, MMA = methyl methacrylate, e-Cap = e-Caprolactam, Nap = Naphthalene, Benz = Benzene, 1-Me-Nap = 1-methyl-naphthalene, 2-Me-Nap = 2-methyl-naphthlaene, C10 ene = C10 alkene, C12 ene = C12 alkene, C14 ene = C14 alkene, C21 diene = C21 alkadiene.

Table S6: Comparison of three extraction methods for removing PE and PVC interferences. Concentrations are of PE and PVC interferences, as calculated from different pyrolysis products and collected on the **0.7 µm filter.**

		PE				PVC			
		**C10 ene**	**C12 ene**	**C14 ene**	**C21 diene**	**Nap**	**Benz**	**1-Me-Nap**	**2-Me-Nap**
Method 1 (CREON only)	*Blank*	*1.86*	*1.01*	*0.47*	*0.11*	*0.70*	*2.01*	*0.84*	*1.04*
	Blood #1	1.86	2.13	1.96	0.25	0.40	0.67	0.48	0.46
	Blood #2	5.59	7.26	6.82	0.87	0.52	0.30	0.60	0.56
	Blood #3	3.89	4.98	4.83	0.54	0.53	0.29	0.57	0.47
	Blood #4	4.15	4.93	4.52	0.51	0.45	0.58	0.52	0.51
	Blood #5	2.47	3.04	2.58	0.35	0.30	0.34	0.28	0.35
	Avg	***3.59***	***4.47***	***4.14***	***0.50***	**0.44**	**0.44**	**0.49**	**0.47**
	STDEV	***1.47***	***1.99***	***1.94***	***0.24***	**0.10**	**0.18**	**0.13**	**0.08**
Method 2 (Proteinase K only)	*Blank*	*1.24*	*3.13*	*0.38*	*<0.10*	*0.028*	*0.21*	*<0.91*	*0.40*
	Blood #1	4.88	19.80	4.64	1.22	0.39	0.20	1.05	0.62
	Blood #2	6.30	15.03	8.60	12.80	0.73	0.48	1.44	1.26
	Blood #3	5.51	17.82	6.72	5.48	0.55	0.22	1.35	0.98
	Blood #4	11.78	14.42	6.83	10.37	0.56	0.24	1.20	1.17
	Blood #5	7.91	18.68	10.29	20.39	0.67	0.29	1.20	1.24
	Avg	***7.27***	***17.15***	***7.42***	***10.05***	***0.58***	***0.28***	***1.25***	***1.05***
	STDEV	***2.76***	***2.33***	***2.13***	***7.30***	***0.13***	***0.17***	***0.15***	***0.27***
Method 3 (final method)	*Blank*	*1.11*	*0.89*	*0.91*	*0.11*	*0.46*	*1.12*	*0.55*	*0.60*
	Blood #1	2.37	2.92	2.46	0.40	0.48	0.38	0.56	0.50
	Blood #2	4.24	5.20	4.56	0.40	0.51	0.79	0.72	0.66
	Blood #3	3.52	3.55	3.18	0.40	0.61	0.44	0.70	0.65
	Blood #4	3.53	4.29	3.69	0.35	0.57	0.46	0.66	0.62
	Blood #5	2.69	3.17	2.98	0.51	0.51	0.27	0.50	0.49
	Avg	***3.27***	***3.82***	***3.37***	***0.41***	***0.53***	***0.47***	***0.63***	***0.58***
	STDEV	***0.75***	***0.93***	***0.80***	***0.057***	***0.051***	***0.19***	***0.091***	***0.082***

Table S7: Comparison of three extraction methods for removing PE and PVC interferences. Concentrations are of PE and PVC interferences, as calculated from different pyrolysis products and collected on the **0.3 µm filter.**

		PE				PVC			
		**C10 ene**	**C12 ene**	**C14 ene**	**C21 diene**	**Nap**	**Benz**	**1-Me-Nap**	**2-Me-Nap**
Method 1 (CREON only)	*Blank*	*0.38*	*0.25*	*0.23*	*0.05*	*0.42*	*1.08*	*0.51*	*0.59*
	Blood #1	1.39	1.59	1.51	0.13	0.43	0.56	0.52	0.53
	Blood #2	2.57	3.29	3.20	0.16	0.54	0.21	0.56	0.47
	Blood #3	2.12	2.86	2.61	0.18	0.50	0.71	0.59	0.71
	Blood #4	1.56	1.98	1.78	0.14	0.34	0.72	0.47	0.47
	Blood #5	1.77	2.31	2.14	0.11	0.54	0.80	0.58	0.71
	Avg	***1.88***	***2.41***	***2.25***	***0.15***	***0.47***	***0.61***	***0.54***	***0.58***
	STDEV	***0.47***	***0.68***	***0.67***	***0.023***	***0.085***	***0.23***	***0.051***	***0.12***
Method 2 (Proteinase K only)	*Blank*	*1.94*	*6.79*	*1.80*	*<0.10*	*<1.29*	*10.9*	*2.32*	*7.17*
	Blood #1	1.46	7.00	0.50	0.77	10.9	2.32	7.17	10.4
	Blood #2	1.83	8.31	1.23	0.15	0.21	0.087	0.28	0.43
	Blood #3	1.83	9.35	0.92	0.12	0.38	0.13	0.24	0.97
	Blood #4	2.17	11.6	0.96	0.14	1.22	0.30	0.78	1.78
	Avg	**1.85**	**8.60**	**1.08**	**0.29**	**2.56**	**0.59**	**1.69**	**2.83**
	STDEV	**0.26**	**1.95**	**0.48**	**0.31**	**4.68**	**0.98**	**3.07**	**4.29**
Method 3 (final method)	*Blank*	*0.58*	*0.56*	*0.58*	*0.022*	*0.30*	*0.90*	*0.34*	*0.39*
	Blood #1	2.43	2.99	2.86	0.25	0.39	0.60	0.51	0.48
	Blood #2	2.44	3.05	2.84	0.18	0.35	0.69	0.48	0.49
	Blood #3	2.81	3.47	3.16	0.29	0.39	0.66	0.55	0.57
	Blood #4	2.70	3.38	3.22	0.16	0.43	0.57	0.61	0.51
	Blood #5	2.62	3.25	2.92	0.22	0.35	0.67	0.48	0.50
	Avg	***2.60***	***3.23***	***3.00***	***0.22***	***0.38***	***0.63***	***0.53***	***0.51***
	STDEV	***0.16***	***0.21***	***0.18***	***0.050***	***0.031***	***0.051***	***0.055***	***0.037***

Table S8: PS and PMMA and COOH-PS nanosphere recoveries from spiked blood samples, presented as range (mean) for different sized nanospheres and calculated using either d_5-PS or 4Fl-PS internal standards. Also listed is the % recovery captured on the 0.3 µm filter (% 0.3 – 0.7 µm sized particles).

**PS**				**PMMA**				**COOH-PS**			
Size (nm)	*%Rec (d5-PS)*	*%Rec (4-FlPS)*	*% 0.3-0.7µm size range*	*Size (nm)*	*%Rec (d5-PS)*	*%Rec (4-FlPS)*	*% 0.3-0.7µm size range*	*Size (nm)*	*%Rec (d5-PS)*	*%Rec (4-FlPS)*	*% 0.3-0.7µm size range*
200 (n=3)	7.8-20 (13)	7.4-17 (12)	91-95%	400 (n=7)	7.3-21 (13)	8.5-19 (12)	21-99%	750 (n=7)	41-72 (52)	39-71 (52)	0%
300 (n=6)	5.2-21 (12)	8.4-30 (19)	42-97%	740 (n=5)	1-28 (11)	1-24 (10)	0-8%				
400 (n=7)	8.7-20 (13)	12-26 (18)	7.4-34%	1100 (n=2)	16-26 (21)	16-29 (22)	0-4%				
500 (n=4)	13-20 (15)	14-21 (17)	19-33%								
700 (n=5)	10-24 (17)	10-24 (17)	0-7%								
1000 (n=2)	15-22 (19)	15-25 (20)	0-10%								

Table S9: Microplastic recoveries (% Rec) from spiked blood samples, presented as range (mean) for different size nanospheres, calculated using either d_5-PS or 4Fl-PS internal standards. Note 100% of all MPs were collected on the 0.7 µm filter.

	PE (C10 alkene)		PP		PS (dimer)		PS (trimer)		PMMA		PC		N-6		N-6,6	
	d_5-PS	4-FlPS	d_5-PS	4-FlPS	d_5-PS	4-FlPS	d_5-PS	4-FlPS	d_5-PS	4-FlPS	d_5-PS	4-FlPS	d_5-PS	4-FlPS	d_5-PS	4-FlPS
% Rec (n=4)	50-65 (57)	55-66 (62)	73-85 (81)	78-100 (88)	25-35 (30)	18-24 (21)	24-39 (31)	18-26 (21)	11-17 (14)	8-11 (9)	1-22 (7)	1-16 (5)	93-116 (109)	98-134 (119)	29-35 (32)	31-41 (35)

	PET (Benzoic acid)		PET (Vinyl benzoate)		PET (Benzophenone)		PVC (Naphthalene)		PVC (Benzene)		PVC (1-Me-Nap)		PVC (2-Me-Nap)	
	d_5-PS	4-FlPS	d_5-PS	4-FlPS	d_5-PS	4-FlPS	d_5-PS	4-FlPS	d_5-PS	4-FlPS	d_5-PS	4-FlPS	d_5-PS	4-FlPS
% Rec (n=4)	27-75 (54)	34-86 (58)	7-16 (11)	8-22 (13)	8-12 (11)	9-13 (12)	11-14 (12)	12-14 (13)	12-26 (19)	13-29 (21)	15-18 (17)	17-21 (18)	20-22 (21)	21-26 (23)

Table S10: Recoveries of PE and d_4-PE as calculated using 21 different pyrolysis products, from spiked blood samples. Data is presented as range (mean) for different size nanospheres. Note 100% of all MPs were collected on the 0.7 µm filter.

**PE**	C10			C12			C14			C18			C19			C20			C21		
	ane	ene	diene	ane	ene	diene	ane	ene	diene	ane	ene	diene	ane	ene	diene	ane	ene	diene	ane	ene	diene
% Rec (n=4)	32-69 (49)	50-65 (57)	37-56 (47)	42-70 (56)	58-77 (67)	53-65 (59)	64-90 (72)	55-77 (64)	35-57 (50)	43-84 (71)	48-62 (54)	38-55 (46)	39-76 (61)	46-58 (52)	38-51 (44)	36-54 (41)	41-56 (47)	35-46 (40)	31-53 (41)	42-57 (49)	37-52 (45)
**d_4-PE**	d_4-C10			d_4-C12			d_4-C14			d_4-C18			d_4-C19			d_4-C20			d_4-C21		
	ane	ene	diene	ane	ene	diene	ane	ene	diene	ane	ene	diene	ane	ene	diene	ane	ene	diene	ane	ene	diene
% Rec (n=4)	46-77 (58)	40-71 (53)	42-81 (59)	27-54 (46)	50-82 (63)	45-74 (56)	44-66 (52)	46-67 (59)	42-77 (57)	48-57 (52)	40-75 (55)	32-65 (45)	60-64 (61)	33-57 (45)	32-67 (46)	31-76 (49)	31-65 (46)	35-66 (48)	21-47 (38)	31-61 (46)	33-53 (40)

Figure S2: Ratios of the calculated PE concentration using different pyrolysis products to the calculated concentration using the C10 alkene, for the pilot study blood samples. The red shading indicates an expected ratio range if PE were present and not an interference. Also presented for comparison are ratios obtained from positive controls, blood samples spiked with either PE or d_4-PE.

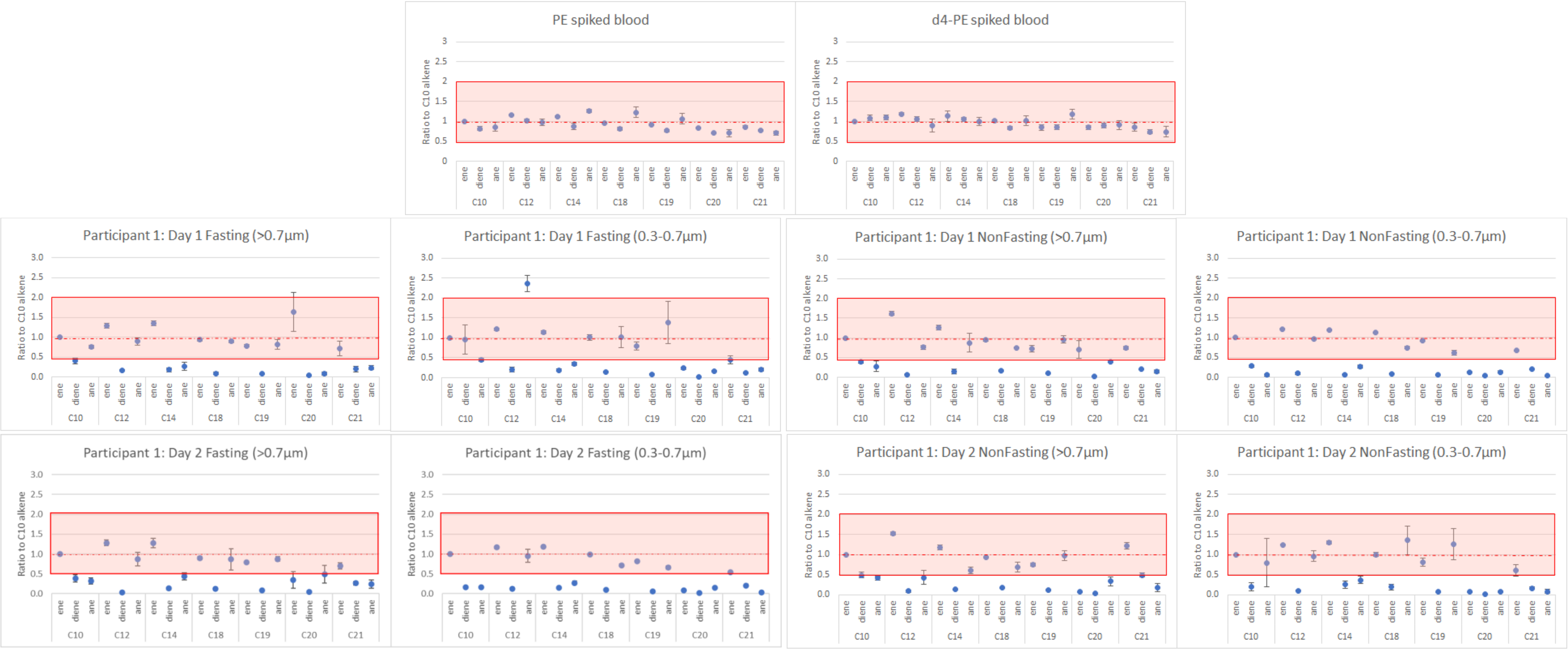

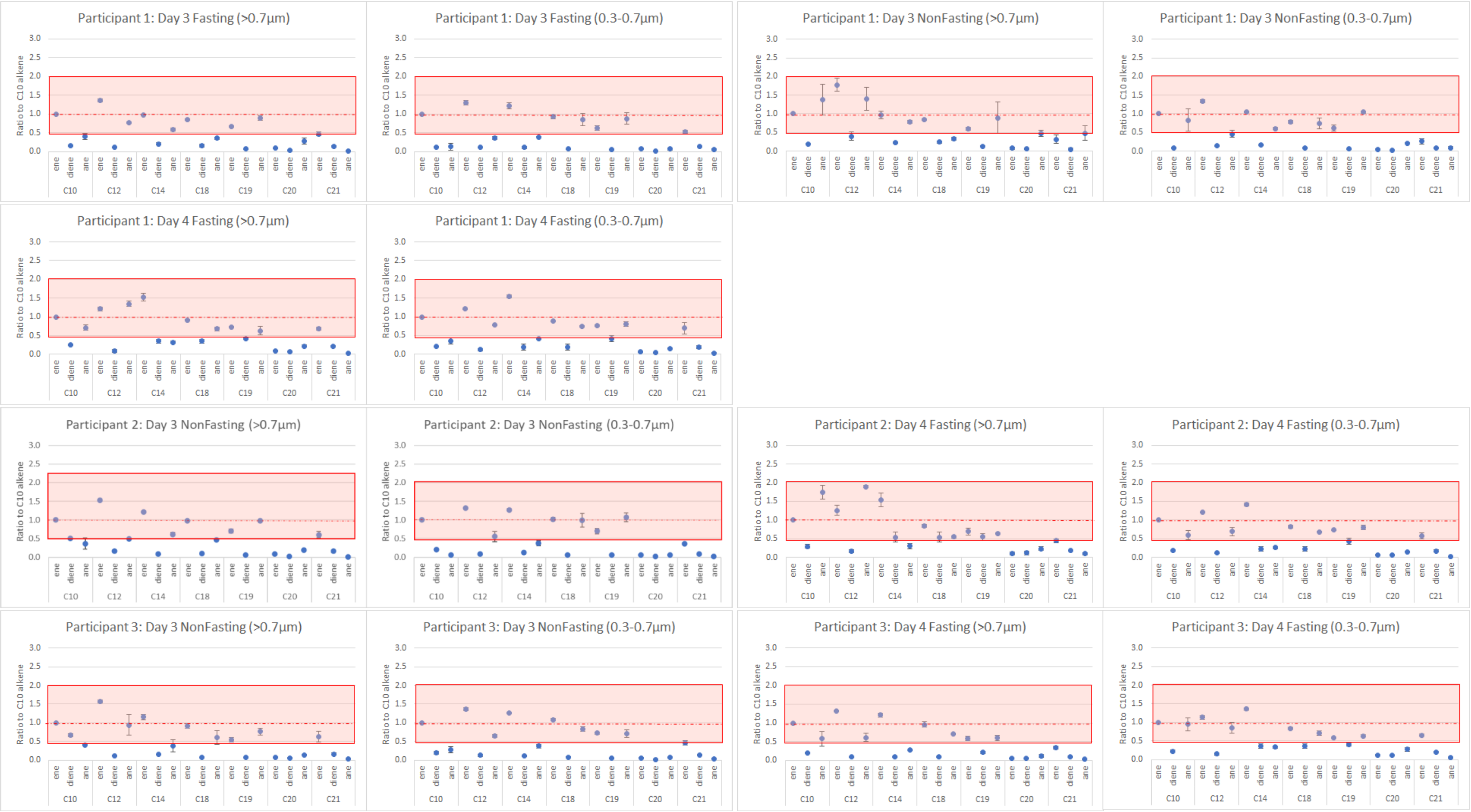
Participant 1: Day 3 Fasting (>0.7µm)
Participant 1: Day 3 Fasting (0.3-0.7µm)
Participant 1: Day 3 NonFasting (>0.7µm)
Participant 1: Day 3 NonFasting (0.3-0.7µm)
Participant 1: Day 4 Fasting (>0.7µm)
Participant 1: Day 4 Fasting (0.3-0.7µm)
Participant 2: Day 3 NonFasting (>0.7µm)
Participant 2: Day 3 NonFasting (0.3-0.7µm)
Participant 2: Day 4 Fasting (>0.7µm)
Participant 2: Day 4 Fasting (0.3-0.7µm)
Participant 3: Day 3 NonFasting (>0.7µm)
Participant 3: Day 3 NonFasting (0.3-0.7µm)
Participant 3: Day 4 Fasting (>0.7µm)
Participant 3: Day 4 Fasting (0.3-0.7µm)
Ratio to C10 alkene
0.0
0.5
1.0
1.5
2.0
2.5
3.0
ene
diene
ane
C10
C12
C14
C18
C19
C20
C21

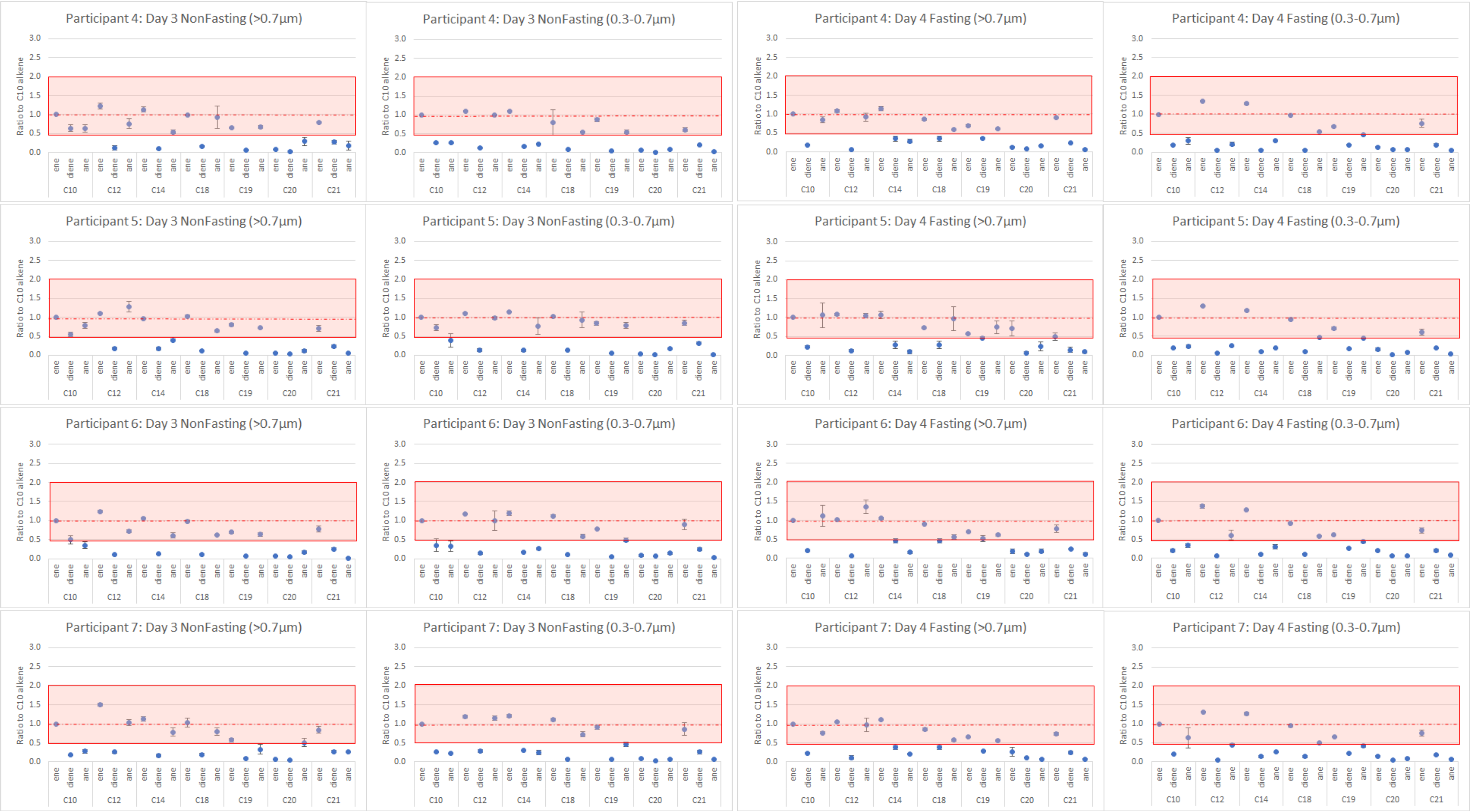

Participant 4: Day 3 NonFasting (>0.7μm)
Participant 4: Day 3 NonFasting (0.3-0.7μm)
Participant 4: Day 4 Fasting (>0.7μm)
Participant 4: Day 4 Fasting (0.3-0.7μm)
Participant 5: Day 3 NonFasting (>0.7μm)
Participant 5: Day 3 NonFasting (0.3-0.7μm)
Participant 5: Day 4 Fasting (>0.7μm)
Participant 5: Day 4 Fasting (0.3-0.7μm)
Participant 6: Day 3 NonFasting (>0.7μm)
Participant 6: Day 3 NonFasting (0.3-0.7μm)
Participant 6: Day 4 Fasting (>0.7μm)
Participant 6: Day 4 Fasting (0.3-0.7μm)
Participant 7: Day 3 NonFasting (>0.7μm)
Participant 7: Day 3 NonFasting (0.3-0.7μm)
Participant 7: Day 4 Fasting (>0.7μm)
Participant 7: Day 4 Fasting (0.3-0.7μm)
Ratio to C10 alkene
0.0
0.5
1.0
1.5
2.0
2.5
3.0
ene
diene
ane
C10
C12
C14
C18
C19
C20
C21

Table S11: Concentrations of MNPs reported in previous human exposure studies employing Py-GC-MS analysis

Reference	Year	Matrix (Human)	Sample Digestion	Instrumentation	Polymers Reported	Particle Size
[1]	2022	Venous Blood	Proteinase K	Frontier EGA/PY-3030D Pyrolysis (double shot)<br>Agilent GCMS	PE: <0.61 – 7.1 µg/mL<br>PET: <0.13 – 2.4<br>PS: <0.34 – 4.8<br>PP: <0.68 – 1.1<br>PMMA: <0.09 – 0.36	≥700 nm
[4]	2023	Faeces	HNO_3	Frontier EGA/PY-3030D Pyrolysis<br>Shimadzu GCMS	PE: 208.1 ± 88.7 µg/g d.w.<br>PVC: 321 ± 238.6<br>PET: 406.7 ± 258.9<br>N6: 15.3 ± 5.6	Not specified
[5]	2023	Semen	HNO_3	Frontier EGA/PY-3030D Pyrolysis<br>Shimadzu GCMS	Sum MNPs: 15.34 ± 23.31 µg/mL	22 – 287µm
[6]	2024	Placenta	10% KOH	Frontier EGA/PY-3030D Pyrolysis<br>Agilent GCMS	Sum MNPs: 126.8 ± 147.5 µg/g<br>PE: 68.8 ± 93.9<br>PVC: 12.9 ±15.9<br>PET: 5.42 ± 6.9<br>PS: 1.19 ± 3.8<br>PP: 1.35 ± 3.58<br>PMMA: 1.98 ± 5.23<br>PC: 1.76 ± 3.14<br>N6: 5.23 ± 6.01<br>N66: 13.4 ± 16.6<br>ABS: 5.65 ± 11<br>PU: 1.84 ± 3.97<br>SBR: 7.15 ± 9.13	Not specified
[7]	2024	Formalin Archived Testes Tissue	10% KOH	Frontier EGA/PY-3030D Pyrolysis<br>Agilent GCMS	Sum MNPs: 161 – 696 µg/g<br>PE: 115.75 ± 94<br>PVC: 9.3 ± 6.09<br>PET: 34.35 ± 17.17<br>PS: 27.5 ± 13.69<br>PP: 18.12 ± 17.23<br>PMMA: 6.75 ± 4.81<br>PC: 28.4 ± 37.5<br>N6: 6.01 ± 4.12	Not specified

					N66: 34.04 ± 16.28<br>ABS: 33.62 ± 17.50<br>PU: 15.17 ± 6.86	
[8]	2024	Arterial Tissue	HNO_3	Frontier EGA/PY-3030D Pyrolysis<br>Shimadzu GCMS	Sum MNPs: 118.66 ± 53.87 µg/g<br>PE: <MDL – 3.44<br>PVC: <MDL – 27.61<br>PET: <MDL – 81.57<br>N66: <MDL – 30.33	Not specified
[9]	2024	Arterial Plaque	None	Frontier EGA/PY-3030D Pyrolysis<br>Agilent GCMS	PE: 21,700 ± 24,500 µg/g<br>PVC: 5,200 ± 2,400	Approx. ≤1 µm
[10]	2024	Thrombi	HNO_3	Frontier EGA/PY-3030D Pyrolysis<br>Shimadzu GCMS	Sum MNPs: 14.88 – 221.8 µg/g<br>*Ischemic Stroke:*<br>PE: 77.0 ± 22.2<br>PVC: 59.6 ± 42.9<br>N66: 57.4 ± 29.2<br>*Myocardial Infarction:*<br>PE: 134.3<br>PVC: 14.4<br>N66: 67.5 ± 25.7<br>*Deep Vein Thrombosis:*<br>N66: 66.4 ± 24.1	20 – 500 µm
[11]	2024	Gallstone	Solvent extraction	Frontier EGA/PY-3030D Pyrolysis<br>Shimadzu GCMS	PE: 3.56 ± 3.21 µg/g<br>PET: 0.18 ± 0.38<br>PS: 10.69 ± 10.56<br>PP: 0.09 ± 0.14<br>EVA: 0.04 ± 0.08	20 – 500 µm
[12]	2024	Vitreous Humor	HNO_3	Frontier EGA/PY-3030D Pyrolysis<br>Shimadzu GCMS	Sum MNPs: 9.46 – 15.73 µg/g (median per disease state)<br>PVC: <MDL – 0.67<br>PS: 0.7 – 1.38<br>N66: 9.14 – 14.76	Not specified
[13]	2024	Blood	As in [1]	Frontier EGA/PY-3030D Pyrolysis (double shot)<br>Agilent GCMS	Sum MNPs: 0.17 – 2.49 µg/mL<br>PE: 0.270 – 1.87<br>PVC: <0.25 – 0.750<br>PET: <0.048 – 0.272<br>PS: <0.036<br>PMMA: <0.031 - 0.052	>300nm

[14]	2024	Sperm	HNO_3	Frontier EGA/PY-3030D Pyrolysis Shimadzu GCMS	PS: 3.57 ± 0.32 µg/mL	>220nm
[15]	2024	Tumor tissue	KOH	Frontier EGA/PY-3030D Pyrolysis Shimadzu GCMS	Sum MNPs: 0.0071 – 0.546 µg/g<br>PE: 0.087 ± 0.117<br>PVC: 0.052 ± 0.082<br>PS: 0.060 ± 0.089	Not specified
[16]	2024	Bone marrow	Various solvent extractions	Frontier EGA/PY-3030D Shimadzu GCMS	Sum MNPs: 15.37 – 92.05 µg/g<br>PE: <MDL – 52.57<br>PVC: <MDL – 40.46<br>PS: 0.33 – 17.26<br>PP: <MDL - 1.75<br>N66: <MDL – 17.18	Not specified
[17]	2024	Urine	Various solvent extractions	Frontier EGA/PY-3030D Shimadzu GCMS	Sum MNPs: <MDL – 7.38 µg/g<br>PE: <MDL – 7.15<br>PVC: <MDL – 0.25<br>N66: <MDL – 0.02	Not specified
[18]	2024	Testes, Semen, Epididymal Cyst	HNO_3	Frontier EGA/PY-3030D Shimadzu GCMS	Sum MNPs: 893 – 9033 µg/g and <MDL – 106 µg/mL<br>*Testis*:<br>PE: <MDL – 5510 µg/g<br>PVC: 409 – 2794<br>N66: 273 – 1622<br>*Semen & cyst fluid*:<br>PE: <MDL µg/mL<br>PVC: <MDL – 56.31<br>PS: <MDL – 0.43<br>PMMA: <MDL – 0.25<br>N66: <MDL – 96.44	Not specified

Abbreviations:

MNPs= micro nanoplastics; PE= Polyethylene; PVC= Polyvinyl chloride; PET= Polyethylene terephthalate; PS= Polystyrene; PP= Polypropylene; PMMA= Poly(methyl methacrylate); PC= Polycarbonate; N6= Nylon 6; N66= Nylon 66; ABS= Acrylonitrile butadiene styrene; PU= Polyurethane; SBR= Styrene-butadiene rubber; EVA= Ethylene-acetate copolymer.

References

1. Leslie, H.A., et al., *Discovery and quantification of plastic particle pollution in human blood.* Environment International, 2022. **163**: p. 107199.
2. Rauert, C., et al., *Extraction and Pyrolysis-GC-MS analysis of polyethylene in samples with medium to high lipid content.* Journal of Environmental Exposure Assessment, 2022. **1**(2): p. 13.
3. Tybor, P.T., et al., *Heat denaturation of blood serum proteins measured in saturated sodium chloride.* Journal of Agricultural and Food Chemistry, 1970. **18**(4): p. 629-631.
4. Ke, D., et al., *Occurrence of microplastics and disturbance of gut microbiota: a pilot study of preschool children in Xiamen, China.* eBioMedicine, 2023. **97**: p. 104828.
5. Zhao, Q., et al., *Detection and characterization of microplastics in the human testis and semen.* Science of The Total Environment, 2023. **877**: p. 162713.
6. Garcia, M.A., et al., *Quantitation and identification of microplastics accumulation in human placental specimens using pyrolysis gas chromatography mass spectrometry.* Toxicological Sciences, 2024. **199**(1): p. 81-88.
7. Hu, C.J., et al., *Microplastic presence in dog and human testis and its potential association with sperm count and weights of testis and epididymis.* Toxicological Sciences, 2024. **200**(2): p. 235-240.
8. Liu, S., et al., *Microplastics in three types of human arteries detected by pyrolysis-gas chromatography/mass spectrometry (Py-GC/MS).* Journal of Hazardous Materials, 2024. **469**: p. 133855.
9. Marfella, R., et al., *Microplastics and Nanoplastics in Atheromas and Cardiovascular Events.* New England Journal of Medicine, 2024. **390**(10): p. 900-910.
10. Wang, T., et al., *Multimodal detection and analysis of microplastics in human thrombi from multiple anatomically distinct sites.* eBioMedicine, 2024. **103**: p. 105118.
11. Zhang, D., et al., *Microplastics are detected in human gallstones and have the ability to form large cholesterol-microplastic heteroaggregates.* Journal of Hazardous Materials, 2024. **467**: p. 133631.
12. Zhong, Y., et al., *Revealing new insights: Two-center evidence of microplastics in human vitreous humor and their implications for ocular health.* Science of The Total Environment, 2024. **921**: p. 171109.
13. Brits, M., et al., *Quantitation of micro and nanoplastics in human blood by pyrolysis-gas chromatography–mass spectrometry.* Microplastics and Nanoplastics, 2024. **4**(1): p. 12.
14. Chen, Y., et al., *Occurrence, toxicity and removal of polystyrene microplastics and nanoplastics in human sperm.* Environmental Chemistry Letters, 2024. **22**(5): p. 2159-2165.
15. Zhao, J., et al., *Detection and quantification of microplastics in various types of human tumor tissues.* Ecotoxicology and Environmental Safety, 2024. **283**: p. 116818.
16. Guo, X., et al., *Discovery and analysis of microplastics in human bone marrow.* Journal of Hazardous Materials, 2024. **477**: p. 135266.
17. Song, X., et al., *Micro(nano)plastics in human urine: A surprising contrast between Chongqing's urban and rural regions.* Science of The Total Environment, 2024. **917**: p. 170455.
18. Yang, W., et al., *Atlas and source of the microplastics of male reproductive system in human and mice.* Environmental Science and Pollution Research, 2024. **31**(17): p. 25046-25058.
